# Multilingual social grooming on social media and self-perceived multilingual identity among Chinese university students

**DOI:** 10.3389/fpsyg.2026.1942436

**Published:** 2026-09-23

**Authors:** Peilu Qin, Yujia Lin, Xin Wang

**Affiliations:** 1 College of Communication Science and Art, Chengdu University of Technology, Chengdu, Sichuan, China; 2 School of Journalism and Communication, Sichuan International Studies University, Chongqing, China; 3 School of Journalism and Communication, Chongqing University, Chongqing, China

**Keywords:** authentic pride, multilingual identity, multilingual social grooming, multilingualism, need for uniqueness, social capital, social media

## Abstract

**Background:**

Chinese university students purposefully use English-only and mixed Chinese-English posts, comments, and replies to share experiences, acknowledge others, and maintain ties, and everyday commentary often reads the practice as showing off. Identity scholarship holds that a multilingual identity is claimed through visible social participation, and quantitative research on digital multilingualism has centred on consumption and learning. This study conceptualises the practice as multilingual social grooming and, drawing on self-conscious emotion and social capital theory, asks whether it relates to self-perceived multilingual identity through authentic pride and online bridging social capital, with need for uniqueness moderating the affective process.

**Methods:**

A questionnaire survey of 755 Chinese university students was analysed with a conditional process model and bootstrap confidence intervals.

**Results:**

Multilingual social grooming was positively associated with self-perceived multilingual identity, directly and through both intervening processes; the relational indirect association was larger. The grooming-pride association and its indirect association were stronger at higher need for uniqueness (*f*^2^ = 0.010); an exploratory negative interaction appeared for bridging capital (*f*^2^ = 0.007).

**Conclusion:**

Relational communication in an additional language is a site of multilingual identity work, tied to a multilingual sense of self through the self-appraisal of competent communication and the wider ties it sustains, strongest for students who value distinctiveness. The findings extend multilingual identity research towards everyday media production.

## 1 Introduction

Multilingual life is increasingly conducted on digital platforms. For a large population of Chinese university students, English is no longer encountered only in classrooms and examinations but circulates through the ordinary flow of social media, where captions, posts, comments, and replies are composed, read, and evaluated within seconds (Ren and Wang, 2026; Wang and Wang, 2025). These platforms are not neutral conduits for such communication; they are connective infrastructures whose interfaces, metrics, and recommendation systems govern what becomes visible and to whom, and what circulates is shaped by curation systems whose responsiveness users actively interpret (Eg et al., 2023; Helmond, 2026; Narayan, 2024; Taylor and Choi, 2022; van Dijck, 2013). Language on social media is therefore always language on display: a choice to write in English, or to mix English and Chinese within a single message, is enacted before a semi-public audience and is subject to that audience’s reactions (Androutsopoulos, 2015; Marwick and Boyd, 2011). What people do with their languages in these spaces is consequently bound up with how they are seen by others, and with how they come to see themselves.

In everyday commentary on Chinese platforms, English-only and mixed Chinese-English posts are often read as “showing off,” a display of education and taste performed for an audience. The same post may also be read as skilled participation, as cosmopolitan self-presentation, or as an ordinary language choice. Because these interpretations depend on audience norms and relational context, multilingual posting can make identity claims unusually salient: the same message that expresses one user’s sense of self can strike part of its audience as a bid for status. The accusation leaves open what the behaviour means to the people who perform it. Whether visible multilingual communication is display, and display alone, is an research question of this study.

This question can be situated within a broader tension between belonging and distinctiveness in multilingual settings. Language choice can signal affiliation and can signal difference, and evaluations of others’ language use are seldom neutral, because the languages a person uses index the communities to which they belong and the social categories through which they are perceived (Giles and Johnson, 1987; Tajfel, 1974). Multilingual social media production is therefore a plausible site at which self-presentation, relational participation, and multilingual self-understanding intersect, and a construct is needed that can represent how individuals locate themselves within a multilingual social world.

Multilingual identity is that construct. It refers to an individual’s explicit understanding of themselves as a multilingual person, a holistic sense of self grounded in the entire linguistic repertoire taken as a whole (Shi and Xu, 2026). Multilingual identity is not equivalent to proficiency, nor is it a count of the languages one has studied; a person may claim a multilingual identity while commanding their languages unevenly and using them in different domains (Block, 2007; Li, 2018). It is, above all, self-perceived and self-relevant: what matters is how individuals position themselves in relation to their languages and what that positioning means for who they take themselves to be and to be becoming (Darvin and Norton, 2015; Fisher et al., 2020). Because identity is negotiated in and through social participation, and is never fixed in advance (Norton, 2013; Norton Peirce, 1995), the repeated, visible use of an additional language in social life is one activity through which a multilingual identity may be claimed, negotiated. The phenomenon of multilingual posting is therefore an identity question: the practice is a site at which a multilingual self is enacted before others.

To connect the practice to the construct, the behaviour itself must be characterised in relational terms, and the concept of social grooming supplies the necessary vocabulary. Social grooming originates in the observation that humans, like other primates, sustain their bonds through frequent low-cost acts of attention, with communication serving as the human medium of grooming (Dunbar, 2004). Applied to social media, it denotes the purposeful self-disclosure and responsive interaction through which users initiate and maintain relationships, updates, acknowledgements, comments, replies, and brief supportive exchanges (Lin, 2019; Tufekci, 2008). Recent work has extended the concept to language-related communication, associating the posting and discussion of language experiences with relational resources and positive affect (Wang and Wang, 2025). Building on this, the present study conceptualises multilingual social grooming as the purposeful use of English-only or mixed English-Chinese posts, comments, and replies to express oneself, acknowledge others, and maintain social ties. So defined, the behaviour is a form of relational media production, distinct from language study and from undifferentiated platform activity, and it is this relational, self-presentational quality that makes it a plausible site of identity work.

If multilingual social grooming is bound to multilingual identity, the connection invites explanation, because relational behaviour does not become part of self-understanding of its own accord. Returning to the practical question, what these users gain, the present study proposes two complementary processes. The first is intrapersonal and affective: successful multilingual communication can be appraised as a competent, effortful accomplishment and can thereby be accompanied by authentic pride, a positive self-conscious emotion tied to achievement and self-worth (Tracy and Robins, 2007; Williams and DeSteno, 2008). The second is interpersonal and relational: the same communicative acts can be accompanied by online bridging social capital, the resources embedded in broad, heterogeneous, comparatively weak ties (Granovetter, 1973; Williams, 2006). The two are genuinely distinct returns, one a self-relevant emotion, the other a perceived network resource, and a user may accrue one without the other. Treating authentic pride and online bridging social capital as parallel processes allows the emotional and relational gains of multilingual posting to be theorised separately while both are related to identity. Treating them as parallel intervening variables permits the two indirect associations to be examined separately.

These associations may not be equally pronounced for everyone. Individuals differ in their need for uniqueness, a relatively stable disposition to maintain a sense of personal distinctiveness and to resist pressures toward conformity (Lynn and Harris, 1997; Snyder and Fromkin, 1977). For a person high in this disposition, using an additional language within a predominantly native-language environment may carry added self-expressive significance (Brewer, 1991). Need for uniqueness is therefore introduced as a dispositional moderator of the grooming-pride association, one of several dispositions that may bear on the practice.

Three gaps motivate the study. First, research on digitally mediated multilingualism has examined identity in two main ways, through interpretive accounts of situated online practice (Androutsopoulos, 2015), and through quantitative work centred on consumption and learning, what learners view, study, and are motivated by (Ebadi et al. (2024), Ren and Wang, (2026), and Zou et al. (2025). Relational multilingual production, the everyday use of an additional language to sustain ties, has figured less often as a correlate of how users understand themselves as multilingual. Second, affective and relational correlates of digital language practice have seldom been integrated within a single model of multilingual identity. Third, little is known about whether a general preference for distinctiveness moderates the association between visible multilingual practice and self-conscious emotion. These gaps correspond to three questions: whether multilingual production is associated with multilingual identity, which affective and relational variables accompany that association, and for whom the affective association is strongest.

The present study addresses these questions with a cross-sectional online questionnaire completed by 755 Chinese university students. It operationalises multilingual social grooming, authentic pride, online bridging social capital, need for uniqueness, and self-perceived multilingual identity, and estimates their direct, indirect, and conditional associations within a single conditional process model, with English proficiency, the number of foreign languages studied, and daily social media time as controls.

The study offers two theoretical contributions and one practical contribution. Theoretically, it first reconceptualises a contested everyday behaviour, English-only and mixed Chinese-English relational communication on social media, as multilingual social grooming and connects this form of media production to self-perceived multilingual identity, complementing research centred on media consumption (Ren and Wang, 2026; Shi and Xu, 2026). Second, it brings an affective variable (authentic pride), a relational variable (online bridging social capital), and a dispositional moderator (need for uniqueness) into one associational model (Tracy and Robins, 2007; Williams, 2006). Practically, it offers a framework for interpreting multilingual self-expression as potentially identity-relevant relational communication.

## 2 Literature review and hypotheses development

### 2.1 Multilingual identity

Because the phenomenon under study is ultimately about how individuals understand themselves as language users, multilingual identity is the organising construct of the model, and its conceptualisation warrants careful development. In social-psychological terms, identity is a person’s sense of who they are in relation to others, constituted through participation in social groups and through the comparisons and distinctions that group life entails (Tajfel, 1974; Tajfel and Turner, 1986). Language is among the most powerful resources through which such identities are formed and signalled, because the languages a person uses index the communities to which they belong and the social categories through which they are perceived (Giles and Johnson, 1987). A multilingual identity, on this footing, is not a by-product of knowing several languages but a distinct way of understanding the self; and among the facets of personal growth that multilingual experience affords, self-understanding of this kind figures among the most prominent (Zhang et al., 2026).

Multilingual identity refers specifically to an individual’s explicit sense of themselves as a multilingual person, anchored in the whole of their linguistic repertoire; it is distinct from the separate identities that might attach to each language taken alone (Shi and Xu, 2026). Two properties of the construct are central. First, it is holistic: the multiple languages of a repertoire are understood as belonging together to a single self, so that even an uneven command of them, with different strengths and domains of use, is compatible with a coherent multilingual self-understanding (Block, 2007; Li, 2018). This holistic view accords with contemporary accounts of the multilingual repertoire as an integrated resource that speakers deploy flexibly across situations, drawing on all their languages together, without alternation between sealed systems (Douglas Fir Group, 2016; Li, 2018). Second, it is self-perceived: what defines a multilingual identity is the individual’s own positioning in relation to their languages, not an external tally of proficiency or of languages studied (Fisher et al., 2020; Shi and Xu, 2026). A multilingual identity can accordingly be understood along the dimensions of how individuals evaluate themselves and their languages, how they experience multilingualism in interaction and in society, and the emotions that being multilingual evokes (Fisher et al., 2020).

Although self-perceived, multilingual identity is not formed in private. Identity-oriented language scholarship holds that identity is socially negotiated: it is claimed and contested in interaction, invested in over time, and bound up with the social positions that a language makes available to a speaker (Norton, 2013; Norton Peirce, 1995). On this view, using a language is itself an act of identity, an occasion on which a person takes up, or is denied, a position as a legitimate user of that language, and through which the relation between the self and the language is renegotiated (Darvin and Norton, 2015). It follows that a multilingual identity is claimed and sustained not by the mere possession of languages but by their repeated use in socially consequential settings, where that use is recognised, or refused, by others. Where such use is visible to an audience, as it is on social media, the negotiation of multilingual identity becomes a public matter.

Optimal-distinctiveness theory offers one way to understand why visible language choices can carry different meanings for different observers. Individuals balance needs for belonging and for distinctiveness (Brewer, 1991); in a network where Chinese is dominant, using English may raise a user’s perceived distinctiveness, and the meaning of that distinctiveness varies with audience, platform, and context. Some observers may treat such use as legitimate multilingual participation; others may treat it as unnecessary display, an evaluation grounded in observers’ own identity commitments (Giles and Johnson, 1987; Tajfel and Turner, 1986).

### 2.2 Multilingual social grooming and self-perceived multilingual identity

Explaining how a visible language practice is related to identity requires a characterisation of the practice in relational terms, since not all social media activity performs the same social work. Even superficially similar acts of posting can serve different functions, and contemporary treatments of social media use accordingly distinguish among functionally distinct forms of use and treat activity as multidimensional (Tuck and Thompson, 2024). For a practice defined by its role in sustaining relationships, the pertinent concept is social grooming.

Social grooming derives from the observation that social bonds are maintained by a steady stream of small, low-cost acts of attention. In humans the medium of such maintenance is communication, and in networked environments it takes the form of purposeful self-disclosure and responsive interaction, sharing updates, acknowledging others’ posts, commenting, replying, and offering brief supportive responses, through which relationships are initiated and kept alive (Dunbar, 2004; Lin, 2019; Tufekci, 2008). The defining feature of grooming is functional: what makes an act grooming is that it performs relational work, keeping people mutually visible and generating recurring occasions for reciprocity without requiring intimate or sustained conversation, a quality well suited to the asynchronous, many-to-many structure of social media (Lin, 2019). Grooming so understood has been associated with the resources and satisfactions of social connection (Liu and Yeo, 2024), and its logic has recently been carried into language-related communication, where posting and discussing language experiences has been associated with relational resources and positive affect (Wang and Wang, 2025).

The present study extends this logic to multilingual communication, defining multilingual social grooming as the purposeful use of English-only or mixed English-Chinese posts, comments, and replies to express oneself, acknowledge others, and maintain social ties. So defined, the construct denotes relational media production: it concerns the visible production of, and response to, multilingual communication in the service of relationships, and is thereby distinguished both from the private study of language and from undifferentiated platform activity (Wang and Wang, 2025). Because mixed communication retains the native and societal language alongside the additional one, multilingual social grooming enacts a repertoire, with no substitution of one language for another; this is what makes it a plausible vehicle for a holistic multilingual identity. Three specifications define the construct. It is behavioural, a matter of concrete communicative acts and their frequency. It is functional, since an act counts through the relational work it performs, such as self-disclosure, acknowledgement, response, and maintenance, the defining feature that social grooming theory supplies (Dunbar, 2004; Lin, 2019). And it is medial, since the acts are performed in an additional language or in mixed code, so that the medium renders the communicator visible as multilingual and ties relational maintenance to language.

These specifications locate the construct among neighbouring concepts. Translanguaging theorises how a speaker deploys an integrated repertoire within an act of meaning-making (Li, 2018). Multilingual social grooming classifies whole acts by the relational work they perform, and it leaves the internal deployment of the repertoire to that literature. Language investment locates a learner’s commitment to the learning enterprise in identity, capital, and ideology (Darvin and Norton, 2015). Multilingual social grooming concerns everyday relational communication conducted in the language. Discourse-analytic accounts of networked multilingualism interpret identity performance within particular online exchanges (Androutsopoulos, 2015). Multilingual social grooming records one function of that practice, relationship maintenance, as an individual-difference variable. Foreign language social grooming counts acts in which the additional language is their topic, such as study logs and practice clips (Wang and Wang, 2025). In multilingual social grooming the additional language is the medium of the acts, their topics span ordinary relational life, and the outcome is multilingual self-perception. Multilingual social grooming may be positively associated with self-perceived multilingual identity because it unites the two conditions that identity theory associates with a consolidated identity: authentic use and social recognition. Grooming is performed before, or for, an audience, so that the user who exercises a language also appears as a multilingual communicator, presenting a multilingual version of the self to others (Goffman, 1959). Because that audience on social media is broad, only partly known, and collapsed into a single imagined public, each act of multilingual grooming is a self-presentation whose reception, through replies, acknowledgement, and continued exchange, supplies evidence about whether a multilingual self-presentation is recognised and sustained (Marwick and Boyd, 2011). And grooming captures the use of language to maintain relationships, something sheer posting frequency does not, embedding multilingual practice in ongoing social life. Users who groom multilingually more often should therefore report more experiences of enacting, and being recognised in, a multilingual role, and a stronger multilingual self-understanding should accompany such experience (Norton, 2013; Shi and Xu, 2026). Accordingly, the following hypothesis is proposed:

- *H1*. Multilingual social grooming is positively associated with self-perceived multilingual identity.

### 2.3 The intrapersonal process: authentic pride

The first process proposed to connect multilingual social grooming to multilingual identity is intrapersonal and affective, turning on the self-conscious emotion of pride. Pride belongs to the family of self-conscious emotions, which arise when people evaluate an event in relation to their standards, goals, and sense of self, and which serve to inform the self about the personal significance of what has occurred (Tracy and Robins, 2004). Pride specifically follows the appraisal that a valued, positive event is attributable to the self, marking an accomplishment as one’s own and as meriting satisfaction (Tracy and Robins, 2004).

Crucially, pride is not unitary. It comprises two conceptually distinct facets: authentic pride, rooted in specific accomplishments and in the appraisal that success reflects one’s effort and competence; and hubristic pride, rooted in global attributions to fixed, superior ability and expressed as arrogance and self-aggrandisement (Tracy and Robins, 2007). The present model concerns authentic pride alone, because the process it describes involves satisfaction with effortful, competent communication, without any sense of inherent superiority, a distinction that will also prove essential when the moderating disposition is introduced, since valuing one’s own distinctiveness must not be conflated with disparaging others. Understood in the language domain, pride of the authentic kind is theorised to arise when learners perceive improvement in their own ability, when their competent use is recognised by others, and when they are able to help others through their language ability (Khajavy and Lüftenegger, 2025; Sulis et al., 2025).

Multilingual social grooming should be positively associated with authentic pride because it repeatedly involves the appraisals with which such pride is linked. Producing a post or reply in an additional language requires the speaker to mobilise their linguistic repertoire in a real communicative situation, and a message that succeeds in expressing what was intended can be appraised as evidence of one’s own effort and competence (Tracy and Robins, 2007). Because grooming is social, that success is not private: acknowledgement and response render it visible, supplying the recognition that authentic pride draws upon, while assisting others through one’s language ability supplies a further occasion for it (Khajavy and Lüftenegger, 2025). Importantly, these appraisals do not require linguistic perfection; authentic pride follows perceived improvement and meaningful participation, with no requirement of flawless performance, so that the ordinary successes of multilingual grooming can be accompanied by it (Williams and DeSteno, 2008).

Authentic pride should, in turn, be positively associated with multilingual identity, because the information that self-conscious emotions carry is precisely information about the self. Authentic pride marks a multilingual accomplishment as both one’s own achievement and personally valued, and positive emotions of this kind are theorised to build enduring personal resources over time, broadening momentary experiences into lasting elements of self-understanding (Fredrickson, 2001; Tracy and Robins, 2004). When pride recurrently accompanies multilingual communication, the accumulated episodes become interpretable as evidence of a valued multilingual capacity that belongs to who one is. Consistent with this reasoning, pride in one’s languages has been associated with a stronger ideal second-language self, an identity-relevant future self-image toward which learners orient (Dörnyei, 2009; Zou et al., 2025).

Pride and identity nonetheless remain distinct, and the proposed process does not conflate them. Authentic pride is an evaluative emotion directed at particular accomplishments; multilingual identity is a broader and more enduring self-understanding in relation to one’s languages (Shi and Xu, 2026; Tracy and Robins, 2007). A person may feel proud of a single successful exchange without regarding multilingualism as central to the self, just as a secure multilingual identity need not be accompanied by pride in every act. The claim, therefore, is that authentic pride is one variable through which repeated multilingual grooming carries self-relevance. Accordingly, the following hypothesis is proposed:

- *H2*. Multilingual social grooming has a positive indirect association with self-perceived multilingual identity through authentic pride.

### 2.4 Online bridging social capital

The second process is interpersonal and relational, turning on the social resources that multilingual grooming can make available. Social capital refers to the resources, information, support, and a sense of connection that individuals can access through their web of social relationships (Putnam, 2000; Williams, 2006). A long-standing distinction separates two forms of such capital: bonding capital, lodged in close, emotionally intense ties and providing deep support; and bridging capital, lodged in broad, heterogeneous, and comparatively weak ties and providing access to novel information and diverse perspectives (Putnam, 2000; Williams, 2006). The present model concerns online bridging capital, because the ties activated by public and semi-public grooming characteristically extend beyond one’s closest relationships.

That bridging resources should reside in weak ties follows from the classic insight that weak ties, by connecting otherwise separate social circles, are the conduits through which non-redundant information and novel perspectives travel (Granovetter, 1973). Social grooming is a principal means of maintaining precisely these ties: its small, recurrent acts of attention keep dispersed and loosely connected contacts within reach, sustaining access to people and information that closer but more homogeneous relationships cannot supply (Ellison et al., 2007; Lin, 2019). Grooming behaviours have been associated with greater bridging social capital in networked settings (Ellison et al., 2007; Liu and Yeo, 2024), and more intensive language-related grooming has been associated with greater perceived social capital and support (Wang and Wang, 2025).

Multilingual grooming should be particularly consequential for bridging capital because language choice bears on the reach of communication. Posting in English or in mixed English-Chinese renders a message accessible or meaningful to partly different audiences, international peers, interest-based communities, and other multilingual users, whose linguistic practices and experiences differ from those of one’s immediate circle, and interaction across such heterogeneous ties is the very substance of bridging capital (Androutsopoulos, 2015; Eren, 2025; Williams, 2006). No single post creates a durable tie, but more frequent relational use of multilingual communication should be accompanied by greater perceived access to diverse contacts and to the informational and perspectival resources they carry.

Online bridging capital should, in turn, be positively associated with multilingual identity through the exposure, participation, and recognition that heterogeneous networks afford. Contact with a wide range of speakers and multilingual others enlarges the set of contexts in which multilingualism appears normal, valuable, and attainable, and offers recurrent opportunities to participate, and to be recognised, as a legitimate multilingual communicator before varied audiences (Androutsopoulos, 2015; Norton, 2013). Bridging capital is a perceived relational resource, and it represents an interpersonal process alongside the intrapersonal process of authentic pride (Shi and Xu, 2026; Williams, 2006). This separation allows bridging capital to represent an interpersonal process that runs parallel to, without duplicating, the intrapersonal process of authentic pride. Accordingly, the following hypothesis is proposed:

- *H3*. Multilingual social grooming has a positive indirect association with self-perceived multilingual identity through online bridging social capital.

### 2.5 Need for uniqueness

The two processes specify how multilingual social grooming may be related to multilingual identity; the remaining question is for whom the affective process is strongest. The relevant individual difference is the need for uniqueness, a relatively stable disposition to preserve a sense of personal distinctiveness from others and to resist pressures toward uniformity (Lynn and Harris, 1997; Snyder and Fromkin, 1977). Uniqueness theory holds that people seek a moderate degree of distinctiveness, differentiating themselves in ways that remain compatible with social acceptance, and that they differ reliably in how strongly they pursue this differentiation (Brewer, 1991; Snyder and Fromkin, 1977). The disposition concerns how much a person values being distinct, not the particular domain in which distinctiveness is sought.

On social media, where users choose what to present and how, communicative style becomes an available vehicle for such differentiation. Within a predominantly native-language environment, communicating in an additional language is one such vehicle: it can constitute a distinctive way of presenting oneself that sets a user apart from the linguistic majority. What matters here is subjective self-differentiation relative to one’s own reference group, independent of any objective rarity, since the same multilingual post may feel ordinary in one network and distinctive in another; and the disposition implies neither that the additional language is inherently prestigious nor that its users seek superiority over others (Brewer, 1991; Lynn and Harris, 1997). Distinctiveness, on this account, is sought as a form of self-expression compatible with belonging; it is not a claim to rank.

This disposition should moderate the affective association specifically. Authentic pride follows the appraisal that a valued accomplishment reflects one’s own effortful and personally expressive conduct (Tracy and Robins, 2007). For users high in need for uniqueness, successful multilingual grooming can carry two kinds of self-relevance at once: it communicates, and it enacts a valued form of personal distinctiveness. For users low in the disposition, the same grooming may still be accompanied by pride, though with less distinctiveness-based significance. The association between multilingual social grooming and authentic pride should therefore be stronger at higher levels of need for uniqueness. Because this is a claim about the strength of an affective appraisal, the emphasis on the authentic facet of pride is essential: a preference for personal distinctiveness concerns the self-relevance of a competent accomplishment and must not be mistaken for a globally self-aggrandising emotion (Tracy and Robins, 2007). A comparable moderating role for the need for uniqueness has been noted in other digital self-presentation settings, where the association between a distinctive platform practice and users’ engagement with it is stronger among those high in the disposition (Chu et al., 2024). Accordingly, the following hypothesis is proposed:

- *H4*. Need for uniqueness moderates the positive association between multilingual social grooming and authentic pride, such that the association is stronger at higher levels of need for uniqueness.

If the association between multilingual social grooming and authentic pride is stronger for users higher in the need for uniqueness, and if authentic pride is in turn associated with multilingual identity, then the indirect association linking grooming to identity through pride should itself be stronger at higher levels of the disposition. For users who value distinctiveness, multilingual grooming should be more strongly bound to pride, and that pride should carry a correspondingly stronger association with a multilingual self-understanding (Shi and Xu, 2026). No parallel conditionality is proposed for the relational process: a preference for personal distinctiveness bears on the self-relevant meaning of an accomplishment, not on whether grooming sustains heterogeneous weak ties, so the association running through bridging capital is expected to hold regardless of the disposition. Restricting the conditional hypothesis to the affective process keeps the intrapersonal and interpersonal processes analytically distinct. Accordingly, the following hypothesis is proposed:

- *H5*. The positive indirect association between multilingual social grooming and self-perceived multilingual identity through authentic pride is stronger at higher levels of need for uniqueness.

These strands form a single account. A multilingual identity is claimed through participation that others can see and respond to (Darvin and Norton, 2015; Norton, 2013), and multilingual social grooming is that participation, the everyday relational use of an additional language before an audience (H1). Such participation carries self-relevance through two accompanying variables. Authentic pride registers the competent, effortful accomplishment of communicating in the language, an emotion whose information is about the self (Tracy and Robins, 2007) (H2). Online bridging social capital registers the widened, heterogeneous ties that the same communication sustains (Williams, 2006) (H3). Need for uniqueness, the disposition developed above, bears on the affective process, since the value a person places on distinctiveness raises the self-relevance of a distinctive multilingual act (H4, H5). The model links a relational behaviour to a multilingual self-perception through one affective and one relational variable, with the affective process conditioned by a preference for distinctiveness.

## 3 Methods

### 3.1 Participants and procedure

This study used a cross-sectional online questionnaire to examine associations among multilingual social grooming, authentic pride, online bridging social capital, need for uniqueness, and self-perceived multilingual identity among Chinese university students. Ethical approval was granted by the Academic Ethics Committee of the School of Journalism and Communication, Chongqing University (approval no. CQUSJC2026012).

Recruitment and data collection were conducted between 10 May and 10 June 2026 by Hangzhou Ladebaode Education Technology Co., Ltd., a third-party survey company. The company administered the online questionnaire and provided participant compensation. The research team supplied the eligibility criteria, questionnaire, informed-consent materials, and data-quality requirements.

For measures originally available in English, two bilingual researchers independently translated the items into Chinese. A third bilingual researcher, who was blind to the original wording, back-translated the Chinese versions into English. The research team compared the original, translated, and back-translated versions, resolved discrepancies, and revised item wording where needed. The original Chinese items of the self-perceived multilingual identity scale were retained, apart from minor presentation adjustments. The resulting questionnaire was then reviewed by three experts whose backgrounds covered applied linguistics, social media and communication research, and quantitative methods. They evaluated conceptual relevance, wording clarity, contextual appropriateness, and potential construct overlap. Further wording adjustments were made in response to this review, and the final wording was fixed before data collection.

Eligibility required participants to be at least 18 years old, currently enrolled at a Chinese college or university, regular users of at least one social media platform, and users of English or mixed Chinese-English communication at least once during the previous 3 months in a social media post, caption, comment, or public or semi-public reply. Entirely private one-to-one messaging and passive consumption of English-language content were not treated as multilingual social grooming. Potential participants viewed an electronic information sheet and provided informed consent before proceeding.

According to the screening report of the survey company, a total of 785 completed questionnaires underwent data-quality screening. The questionnaire contained one instructed-response attention check; the company excluded the 30 completed questionnaires that failed this check before delivering the data, yielding a final analytic sample of 755 valid responses. The excluded records were not available to the research team. No completion-time threshold was applied. All retained responses were complete, with no missing values on the focal measures. Maximum longstring was examined only in a sensitivity analysis and was not a primary exclusion rule (Appendix Table 18). These screening procedures follow established recommendations for identifying insufficient-effort responding (Dunn et al., 2018; Meade and Craig, 2012).

Of the 755 participants, 435 (57.6%) were women and 320 (42.4%) were men. Ages ranged from 18 to 24 years (M = 20.00, SD = 1.60). Academic disciplines comprised the humanities and social sciences (265, 35.1%), science, technology, engineering, and mathematics (208, 27.5%), business and economics (184, 24.4%), and the arts and sports (98, 13.0%). A total of 374 participants (49.5%) reported having studied one foreign language, English only, and 381 (50.5%) reported having studied two or more. Self-rated English proficiency averaged 4.15 (SD = 1.26) on a seven-point scale. Appendix Table 1 provides the complete sample and control-variable distributions.

Participants were enrolled at institutions in first-tier (172, 22.8%), second-tier (223, 29.5%), third-tier (239, 31.7%) and fourth-tier (121, 16.0%) cities, and 40 participants (5.3%) identified as members of an ethnic minority (Han: 715, 94.7%). Minority participants were represented in every city tier, and their share did not differ by tier (chi-square(3) = 4.75, *p* = 0.191).

### 3.2 Measurements

The questionnaire comprised five focal measures, three control variables, demographic questions, and one instructed-response attention check. Unless otherwise stated, focal items used seven-point response scales, and construct scores were computed as item means. The full wording of every focal item is presented alongside its item-level descriptive statistics in Appendix Tables 2–6, and the eligibility screening items, the instructed-response attention check, and the demographic and control questions are reproduced verbatim in Section 1.4 of the appendices, so a separate duplicated questionnaire is not included.

Multilingual social grooming was assessed using an eight-item, study-specific measure informed by previous operationalisations of online social grooming (Lin, 2019; Liu and Yeo, 2024; Wang and Wang, 2025). The items were contextualised to English-only and mixed Chinese-English communication on social media, including posts, captions, comments, and public or semi-public replies used for self-disclosure, responsive interaction, and relationship maintenance. Contextualisation also specified a three-month reference period and a frequency-based response format. The items were drafted in Chinese and English in parallel by the bilingual research team, cross-language equivalence was checked through back-translation, and the expert review and wording adjustments described in Section 3.1 applied to the final wording. Participants indicated frequency on a seven-point scale from 1 (never) to 7 (daily or almost daily). Item scores were averaged, with higher values indicating more frequent multilingual social-grooming behaviour. Sample items were “I use English or Chinese-English mixed captions or posts to share my daily experiences with other people on social media”, “I use English or Chinese-English mixed comments to respond to other people’s posts on social media” and “I use English or Chinese-English mixed communication to maintain my relationships with other people on social media”; the complete item set is reproduced in Appendix Table 2.

Authentic pride was measured with the seven authentic-pride items of Tracy and Robins (2007), contextualised to participants’ English-only and mixed Chinese-English social media communication during the previous 3 months. Participants indicated how strongly those experiences generally made them feel each emotion on a seven-point scale from 1 (not at all) to 7 (extremely). The score was the mean of the seven items.

Online bridging social capital was measured with nine items adapted from Williams’s (2006) internet bridging social capital subscale. The items assessed perceived access to heterogeneous contacts, unfamiliar perspectives, wider communities, and new relational resources through social media. One behavioural-intention item concerning support for general online community activities was not included to maintain conceptual separation from multilingual social grooming. Participants responded on a seven-point agreement scale. The score was the mean of the nine items. Because the items referred to social media interaction generally, the measure does not isolate social capital arising specifically from multilingual exchanges.

Need for uniqueness was measured with the four-item Self-Attributed Need for Uniqueness Scale of Lynn and Harris (1997). Participants responded on a seven-point agreement scale. The score was the mean of the four items, with higher values indicating a stronger preference for personal distinctiveness.

Self-perceived multilingual identity was measured with the evaluation dimension of the multilingual identity scale developed and validated among Chinese university students by Shi and Xu (2026). The full instrument operationalises the evaluation-experience-emotion framework of Fisher et al. (2024) in three subscales; the evaluation subscale used here comprises 16 items in three factors: evaluation of self (six items; for example, “I see the ability to speak multiple languages as part of my identity”), evaluation of language (six items), and evaluation of culture (four items). The evaluation dimension measures the study’s outcome directly: participants’ explicit appraisal of themselves and their languages, the evaluative self-perception at the centre of the construct. The experience dimension indexes engagement in multilingual practices and encouragement from others, content that belongs on the predictor side of the model, and the emotion dimension indexes learning-related emotions, content that belongs with the affective intervening variable; confining the outcome to the evaluation dimension keeps the three roles of the model distinct. Items were administered in the original Chinese wording on a seven-point agreement scale, extending the source’s six-point format for consistency with the other measures. The three-factor structure reported by Shi and Xu was reproduced in the present data, with the three factors loading on a higher-order factor (Section 4.2); the primary score was the mean of all 16 items, and dimension-level scoring was examined in sensitivity analyses. The outcome is accordingly interpreted throughout as the evaluative component of multilingual identity.

The primary models included self-rated English proficiency, number of foreign languages studied, and daily time on social media. English proficiency was rated from 1 (very low) to 7 (very high). Number of foreign languages studied was recorded as 1 (English only), 2, or 3 (three or more). Daily social media time was coded from 1 (less than 30 min) to 5 (more than 3 h). Age, gender, and academic major were collected for description and entered only in extended-control sensitivity analyses. Ordered control categories were entered as numeric scores in the primary models; dummy-coded sensitivity analysis of the exploratory relational interaction produced the same conclusion (Appendix Table 23). City tier of the institution (first- to fourth-tier city, entered as three dummy variables with first-tier cities as the reference category) and ethnicity (0 = Han, 1 = ethnic minority) were added as controls in the robustness analyses.

### 3.3 Analysis

Analyses were conducted in Python. The analysis code, package versions, random seeds, and bootstrap settings accompany the manuscript and are summarised in Section 8 of the appendices. The source data were not altered.

Measurement evidence was evaluated confirmatorily against conventional fit criteria (Hu and Bentler, 1999). As a preliminary structural check, an exploratory factor analysis of the 44 focal items was conducted with principal axis factoring, varimax rotation, and parallel analysis; sampling adequacy was assessed with the Kaiser-Meyer-Olkin statistic and Bartlett’s test of sphericity, and complete results are reported in Section 7 of the appendices (Appendix Tables 24, 25). For the contextually adapted multilingual social-grooming measure, item means, standard deviations, skewness, kurtosis, corrected item-total correlations, standardised confirmatory loadings, Cronbach’s alpha, composite reliability, and average variance extracted were examined. The identity scale was modelled with three first-order dimensions loading on a higher-order identity factor. Internal consistency was assessed with Cronbach’s alpha and composite reliability. Convergent validity was examined using average variance extracted, and discriminant validity was considered using heterotrait-monotrait ratios (Henseler et al., 2015), Fornell–Larcker comparisons (Fornell and Larcker, 1981), and theoretically relevant alternative confirmatory models. Because these analyses use the same sample as the substantive tests, the eight items were additionally validated in an independent sample (*N* = 220) with a prespecified single-factor confirmatory model, reliability and convergent-validity statistics, comprehension checks, relations with single-item indicators of neighbouring behaviours and proficiency, and gender invariance tests; the procedure and complete results are reported in Appendix Section 9.

Three common-method diagnostics were reported (Podsakoff et al., 2003): Harman’s single-factor percentage, a one-factor confirmatory model, and an unmeasured latent method-factor model.

The prespecified H1 to H5 associations were examined within Hayes’s (2022) conditional process framework, using ordinary least squares regression with authentic pride and online bridging social capital as parallel intervening variables and need for uniqueness moderating the grooming-pride association. Multilingual social grooming and need for uniqueness were mean-centred before forming their interaction. English proficiency, number of foreign languages studied, and daily social media time were included as controls. Because Breusch-Pagan tests indicated heteroscedasticity in all three equations, HC3 standard errors and confidence intervals are used as the primary regression inference. Indirect and conditional indirect associations were evaluated using percentile confidence intervals from 5,000 case-resampling bootstrap samples with a fixed seed.

An exploratory extension added the grooming-by-uniqueness interaction to the online bridging social-capital equation. This interaction was not part of H1 to H5 and is reported as exploratory. Its conditional slopes, the contrast between the affective and relational interaction coefficients, and the moderated indirect index through bridging capital were evaluated with HC3 inference and 10,000 case-resampling bootstrap samples. Robustness analyses examined models without controls, extended controls, an alternative longstring screen, alternative identity scoring, a nonmoderated latent-variable structural model, language-study subgroups, reverse-order regressions, and alternative coding of ordered controls. Robustness analyses also re-estimated the primary model with city tier and ethnicity added as controls, singly and together, and with the full extended set (city tier, ethnicity, age, gender and field of study), using HC3 inference and 5,000-sample percentile bootstrap intervals for the indirect associations. Composite means were compared across city tiers with Welch analysis of variance and between Han and ethnic minority participants with Welch t tests and Hedges’ g, and the smallest standardised difference detectable with 80% power for the ethnicity comparison was computed. Cohen’s *f*^2^ was calculated for every interaction term (Cohen, 1988).

## 4 Results

### 4.1 Descriptive statistics and correlations

On the seven-point metric, composite means were 4.64 (SD = 1.32) for multilingual social grooming, 4.58 (SD = 1.26) for authentic pride, 4.59 (SD = 1.29) for online bridging social capital, 4.42 (SD = 1.37) for need for uniqueness, and 4.51 (SD = 1.12) for self-perceived multilingual identity. Figure 1 displays the five distributions. Item-level statistics are reported with full question wording in Appendix Tables 2–6, and composite statistics are reported in Appendix Table 7.

**Figure 1:**
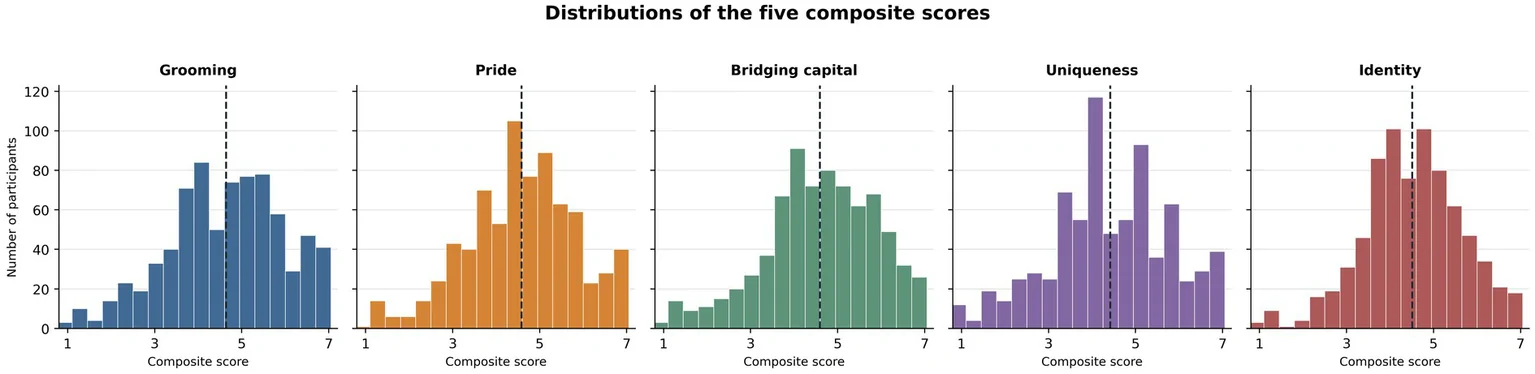
Distributions of the five composite scores. Dashed lines mark the sample means.

As shown in Table 1, all five focal variables were positively intercorrelated (all *p* < 0.001). Multilingual social grooming correlated with self-perceived multilingual identity (*r* = 0.72), authentic pride (*r* = 0.56), and online bridging social capital (*r* = 0.65). Figure 2 visualises the complete correlation matrix, and Appendix Table 14 reports its exact values and significance tests.

**Table 1:** Means, standard deviations, reliabilities, and correlations among the focal variables.

**Variable**	**Mean**	**Standard deviation**	**Cronbach’s alpha**	**1**	**2**	**3**	**4**	**5**
1. Multilingual social grooming	4.64	1.32	0.93					
2. Authentic pride	4.58	1.26	0.90	0.56				
3. Online bridging social capital	4.59	1.29	0.93	0.65	0.50			
4. Need for uniqueness	4.42	1.37	0.89	0.33	0.35	0.31		
5. Self-perceived multilingual identity	4.51	1.12	0.94	0.72	0.69	0.73	0.37	

*N* = 755. All displayed correlations have *p* < 0.001.

**Figure 2:**
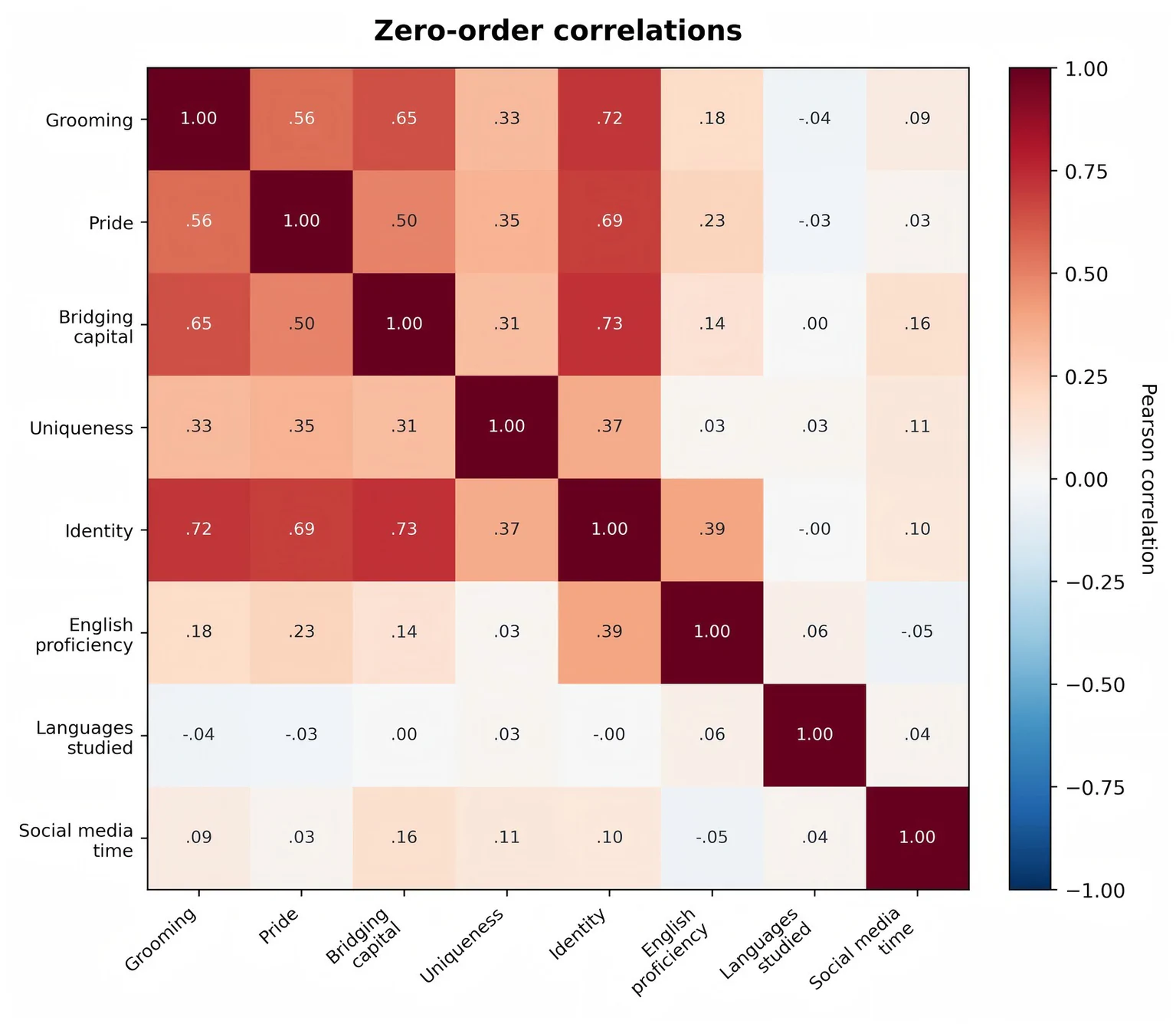
Zero-order correlations among the five focal variables and three control variables (*N* = 755).

### 4.2 Measurement evidence

A preliminary exploratory factor analysis supported the suitability of the 44-item pool for factor analysis (Kaiser-Meyer-Olkin = 0.976; Bartlett’s chi-square(946) = 22,259.5, *p* < 0.001). Parallel analysis indicated that up to six factors could be retained, a pattern consistent with the three-dimensional structure of the identity scale, and in the theoretically specified five-factor solution 34 of the 44 items showed primary loadings of at least 0.40 on their intended constructs, with no cross-loading above 0.39; every item with a lower primary loading belonged to the identity scale (Appendix Tables 24, 25). The higher-order confirmatory specification reported next accommodates this structure.

The higher-order confirmatory model, in which the three identity dimensions loaded on a second-order identity factor within the five-construct model, fit the data well: chi-square(889) = 1,109.25, comparative fit index = 0.990, Tucker-Lewis index = 0.989, and root-mean-square error of approximation = 0.018. The standardised second-order loadings were 0.84 for evaluation of self, 0.81 for evaluation of language, and 0.84 for evaluation of culture. A separate correlated three-factor model of the 16 identity items also fit well: comparative fit index = 0.998, Tucker-Lewis index = 0.998, root-mean-square error of approximation = 0.013, and standardised root-mean-square residual = 0.021. Appendix Table 8 reports these results.

Table 2 summarises reliability and convergent validity. The study-specific multilingual social-grooming items had corrected item-total correlations from 0.70 to 0.78 and standardised loadings from 0.73 to 0.82. Cronbach’s alpha was 0.93, composite reliability was 0.93, and average variance extracted was 0.61. These values provide preliminary evidence of internal consistency and convergent validity in this sample, but independent validation remains necessary because the item set was contextually adapted and evaluated in the same sample as the substantive analyses. The three identity dimensions also showed high internal consistency. Additional reliability details are in Appendix Table 9.

**Table 2:** Reliability and convergent-validity evidence.

Construct or dimension	Number of items	Cronbach’s alpha	Composite reliability	Average variance extracted
Multilingual social grooming	8	0.93	0.93	0.61
Authentic pride	7	0.90	0.91	0.58
Online bridging social capital	9	0.93	0.93	0.61
Need for uniqueness	4	0.89	0.89	0.68
Self-perceived multilingual identity, global score	16	0.94		
Envisioned multilingual self and identity centrality	6	0.90		
Envisioned linguistic and cognitive advantages	6	0.90		
Envisioned intercultural understanding and orientation	4	0.86		

Composite reliability and average variance extracted are reported for the four first-order constructs represented as unidimensional factors. The global identity measure was interpreted through its higher-order three-dimensional model rather than a flat single-factor average-variance estimate.

In the independent sample (*N* = 220), the prespecified single-factor model fit well, scaled chi-square(20) = 24.46, *p* = 0.223, comparative fit index = 0.993, Tucker-Lewis index = 0.990, root-mean-square error of approximation = 0.032, 90% CI [0.000, 0.070], standardised root-mean-square residual = 0.031; standardised loadings ranged from 0.57 to 0.78 with all 95% confidence intervals above 0.40; Cronbach’s alpha was 0.86, 95% CI [0.83, 0.89], composite reliability was 0.86 and average variance extracted was 0.45, which meets Fornell and Larcker's (1981) criterion for convergent validity given composite reliability above 0.60. The single-factor model was invariant across gender, and the grooming score was related to, but not redundant with, overall posting and commenting frequency, browsing of English content, private English messaging and self-rated proficiency (*r* = 0.26 to 0.49). Appendix Section 9 reports the complete validation.

Discriminant-validity evidence was mixed. All heterotrait-monotrait ratios were below 0.85 (maximum = 0.78), but the Fornell–Larcker criterion was not met for several comparisons involving the broad identity construct. Alternative models that collapsed grooming with bridging capital or pride with identity fit materially worse than the distinct-construct model. Appendix Tables 10–12 report the complete matrices and model comparisons. These findings support empirical distinguishability while limiting strong claims of discriminant validity for identity.

### 4.3 Common method variance

Harman’s first unrotated factor accounted for 41.9% of item variance, and a one-factor confirmatory model fit poorly (comparative fit index = 0.688; root-mean-square error of approximation = 0.100). An unmeasured latent method-factor model fit better (comparative fit index = 0.964; root-mean-square error of approximation = 0.035) and produced a mean squared method loading of 0.384. A general factor therefore accounted for substantial covariance, as is expected when five positively related constructs are measured with one instrument, and the diagnostics cannot apportion that covariance between method and substance. The interaction results are informative on this point: common method variance inflates linear associations but attenuates, and cannot generate, interaction effects (Siemsen et al., 2010), so the moderation findings reported below are not attributable to it.

### 4.4 Prespecified H1 to H5 associations

The primary conditional process model included authentic pride and online bridging social capital as parallel intervening variables, need for uniqueness as a moderator of the grooming-pride association, and the three controls. It explained 36.9% of variance in authentic pride, 43.2% in online bridging social capital, and 76.2% in self-perceived multilingual identity. Figure 3 reports control-adjusted standardised coefficients, and Table 3 reports unstandardised focal coefficients with HC3 inference. Full equations, including controls, are in Appendix Table 15; diagnostics are in Appendix Table 16.

**Figure 3:**
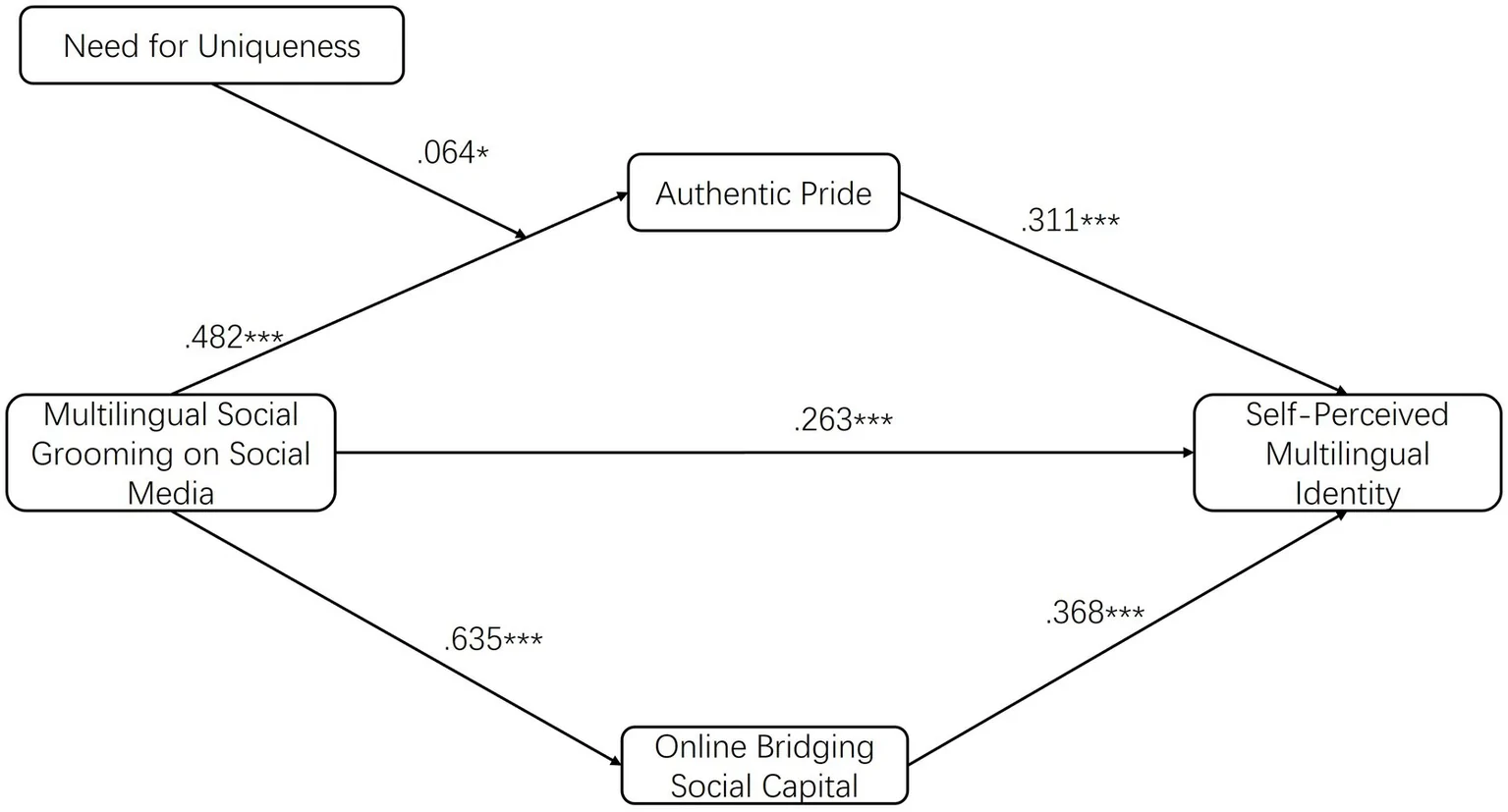
Control-adjusted standardised primary model. Associations involving the control variables are omitted for clarity. The online bridging equation shown here is the prespecified equation without a relational interaction. Asterisks indicate *p* < 0.05 (*) and *p* < 0.001 (***).

**Table 3:** Focal regression coefficients in the primary conditional process model.

Criterion and predictor	Coefficient	HC3 standard error	Test statistic	*p-*value	95% HC3 confidence interval
Authentic pride: grooming	0.461	0.031	14.89	<0.001	[0.400, 0.522]
Authentic pride: need for uniqueness	0.188	0.027	6.85	<0.001	[0.134, 0.242]
Authentic pride: grooming by uniqueness	0.044	0.020	2.16	0.031	[0.004, 0.084]
Online bridging social capital: grooming	0.620	0.028	22.01	<0.001	[0.565, 0.676]
Multilingual identity: grooming	0.224	0.022	9.98	<0.001	[0.180, 0.268]
Multilingual identity: authentic pride	0.277	0.021	13.35	<0.001	[0.236, 0.317]
Multilingual identity: online bridging social capital	0.321	0.022	14.81	<0.001	[0.279, 0.364]

Coefficients are unstandardised. Grooming and need for uniqueness were mean-centred. Models controlled for English proficiency, number of foreign languages studied, and daily social media time.

Multilingual social grooming had a zero-order correlation of 0.718 with self-perceived multilingual identity. The unadjusted total slope was 0.612 (HC3 standard error = 0.023, 95% confidence interval [0.566, 0.658], *p* < 0.001), and the control-adjusted total slope was 0.566 (HC3 standard error = 0.026, 95% confidence interval [0.515, 0.617], *p* < 0.001). With pride and bridging capital included, the direct association remained positive, b = 0.224, HC3 standard error = 0.022, 95% confidence interval [0.180, 0.268], *p* < 0.001. The data were therefore consistent with H1.

Multilingual social grooming was positively associated with authentic pride, which in turn was positively associated with multilingual identity. The indirect association through pride was 0.128, with a 95% bootstrap confidence interval [0.103, 0.153], consistent with H2. Grooming was also positively associated with online bridging social capital, which was positively associated with identity. The indirect association through bridging capital was 0.199, with a 95% bootstrap confidence interval [0.169, 0.232], consistent with H3. A bootstrap contrast indicated that the relational indirect association was larger by 0.072, 95% bootstrap confidence interval [0.032, 0.111]. Table 4 reports the indirect results.

**Table 4:** Indirect and conditional indirect associations in the prespecified model.

Association	Estimate	95% percentile bootstrap confidence interval
Indirect through authentic pride (H2)	0.128	[0.103, 0.153]
Indirect through online bridging social capital (H3)	0.199	[0.169, 0.232]
Index of moderated indirect association through pride (H5)	0.012	[0.002, 0.024]
Conditional pride indirect at low uniqueness	0.111	[0.079, 0.140]
Conditional pride indirect at mean uniqueness	0.128	[0.103, 0.153]
Conditional pride indirect at high uniqueness	0.144	[0.117, 0.174]

*N* = 755. Confidence intervals are based on 5,000 case-resampling bootstrap samples. Low and high uniqueness are one standard deviation below and above the mean.

Need for uniqueness moderated the grooming–pride association, *b* = 0.044, HC3 standard error = 0.020, 95% HC3 confidence interval [0.004, 0.084], *p* = 0.031, consistent with H4; the interaction accounted for 0.006 of incremental variance (*f*^2^ = 0.010, below Cohen's, 1988 benchmark of 0.02 for a small effect). Figure 4 displays the interaction. The conditional grooming-pride slopes were 0.400 at low uniqueness, 0.461 at mean uniqueness, and 0.522 at high uniqueness; all were positive with *p* < 0.001. Figure 5 shows that the HC3 confidence interval remained above zero throughout the observed uniqueness range from 1 to 7.

**Figure 4:**
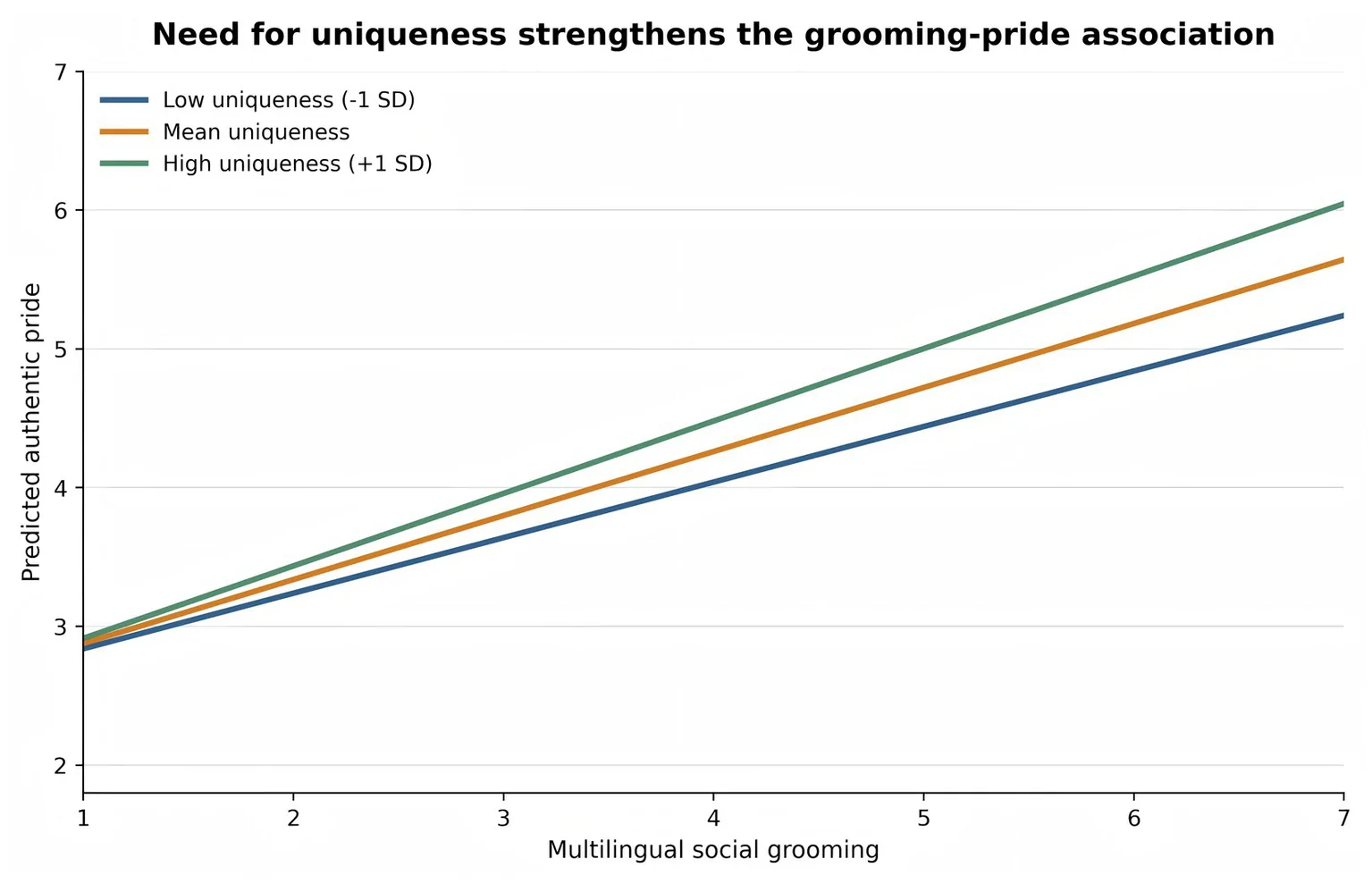
Simple slopes of multilingual social grooming on authentic pride.

**Figure 5:**
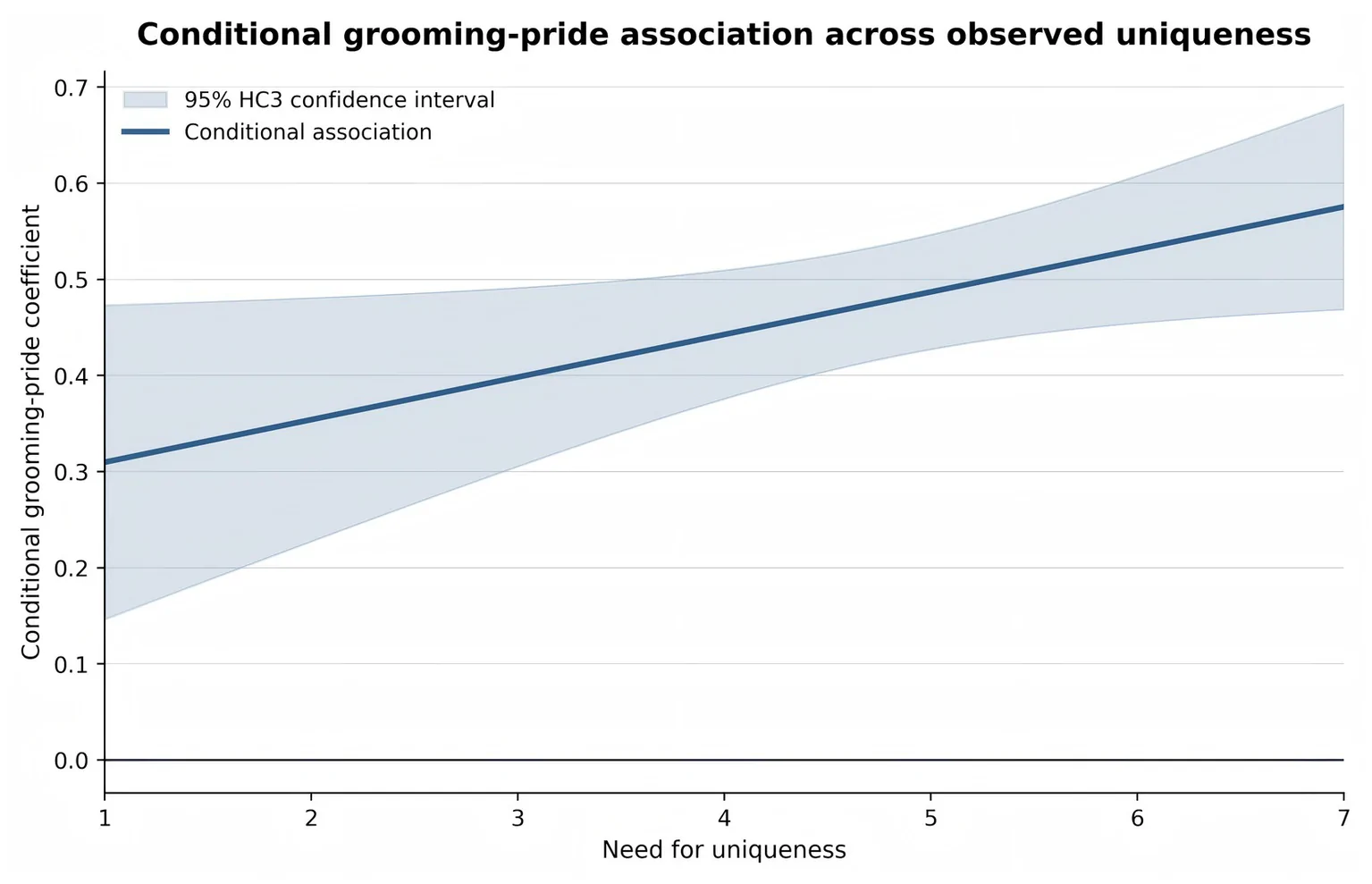
Conditional grooming-pride association across the observed range of need for uniqueness.

The index of the moderated indirect association through pride was 0.012, with a 95% bootstrap confidence interval [0.002, 0.024], consistent with H5. The conditional pride indirect association increased from 0.111 at low uniqueness to 0.144 at high uniqueness (Table 4).

### 4.5 Exploratory relational moderation

To examine whether the relational association also varied with need for uniqueness, an exploratory equation added the same interaction to online bridging social capital. As Table 5 shows, the interaction was small and negative, b = −0.037, HC3 standard error = 0.014, 95% HC3 confidence interval [−0.065, −0.008], *p* = 0.011, with an incremental R-squared of 0.004 (*f*^2^ = 0.007, below the benchmark for a small effect). The grooming-bridging association remained positive at low, mean, and high uniqueness, but declined from 0.633 to 0.532. Appendix Figure 1 visualises these slopes, and Appendix Table 17 gives the expanded results.

**Table 5:** Exploratory moderation of the relational association.

Quantity	Estimate	HC3 standard error	Test statistic	*p*-value	95% confidence interval
Grooming by uniqueness interaction in bridging equation	−0.0367	0.0144	−2.55	0.011	[−0.0650, −0.0084]
Grooming slope at low uniqueness	0.6331	0.0317	20.00	<0.001	[0.5709, 0.6952]
Grooming slope at mean uniqueness	0.5826	0.0286	20.39	<0.001	[0.5265, 0.6386]
Grooming slope at high uniqueness	0.5321	0.0376	14.14	<0.001	[0.4582, 0.6059]
Affective interaction minus relational interaction	0.0810	0.0186	4.36	<0.001	[0.0446, 0.1174]
Moderated indirect index through bridging capital	−0.0118				[−0.0211, −0.0024]

This analysis was not part of H1 to H5. Cohen’s *f*^2^ for the interaction = 0.007; for the affective interaction in Table 3, *f*^2^ = 0.010. The final row uses a 10,000-sample percentile bootstrap confidence interval. A separate 10,000-sample bootstrap interval for the difference between interaction coefficients was [0.0475, 0.1199].

The affective and relational interaction coefficients differed by 0.081, with a 10,000-sample bootstrap confidence interval [0.048, 0.120]. The moderated indirect index through bridging capital was −0.0118, with a 95% bootstrap confidence interval [−0.0211, −0.0024]. Thus, need for uniqueness was associated with a stronger affective association but a modestly weaker relational association. Both first-stage associations remained positive across the observed range.

### 4.6 Robustness and sensitivity analyses

The principal direct and indirect associations were broadly similar without controls, with an extended control set, and after excluding the 11 retained cases with a maximum longstring of 16 or more. The pride interaction remained detectable with HC3 inference in these specifications (*p* = 0.012, 0.022, and 0.017, respectively). Appendix Table 18 reports the corresponding estimates.

Adding city tier and ethnicity as controls left every focal estimate unchanged to the second decimal (Figure 6; Appendix Tables 37, 38). With both added, the grooming–pride coefficient was 0.458 (95% HC3 CI [0.397, 0.519]), the grooming-by-uniqueness interaction 0.042 [0.002, 0.083], the grooming–bridging coefficient 0.617 [0.562, 0.673], and the direct grooming–identity coefficient 0.225 [0.181, 0.270]; the indirect associations were 0.126 [0.102, 0.151] through pride and 0.198 [0.167, 0.231] through bridging capital, and the full extended set produced the same pattern. City tier was not associated with bridging capital or identity (joint HC3 tests *p* = 0.563 and 0.647) and only marginally with pride (*p* = 0.059), where students at second- and third-tier institutions reported slightly higher pride than those at first-tier institutions (b = 0.192 and 0.162). Composite means did not differ across tiers (Welch *F* ≤ 2.45, *p* ≥ 0.063, eta-squared ≤ 0.009; Appendix Table 35 and Appendix Figure 3). Ethnic minority participants did not differ from Han participants on grooming, pride, identity or need for uniqueness (|g| ≤ 0.20); they reported somewhat lower bridging capital (g = −0.25, 95% CI [−0.57, 0.07]) and higher self-rated English proficiency (*g* = 0.36, 95% CI [0.04, 0.68]). As a control, ethnicity was not associated with any outcome (*p* ≥ 0.061), and exploratory grooming-by-ethnicity interactions were all near zero (Appendix Table 36). With 40 minority participants, the comparison could detect only standardised differences of 0.46 or larger with 80% power, so these results rule out large ethnic-group differences, not small ones.

**Figure 6:**
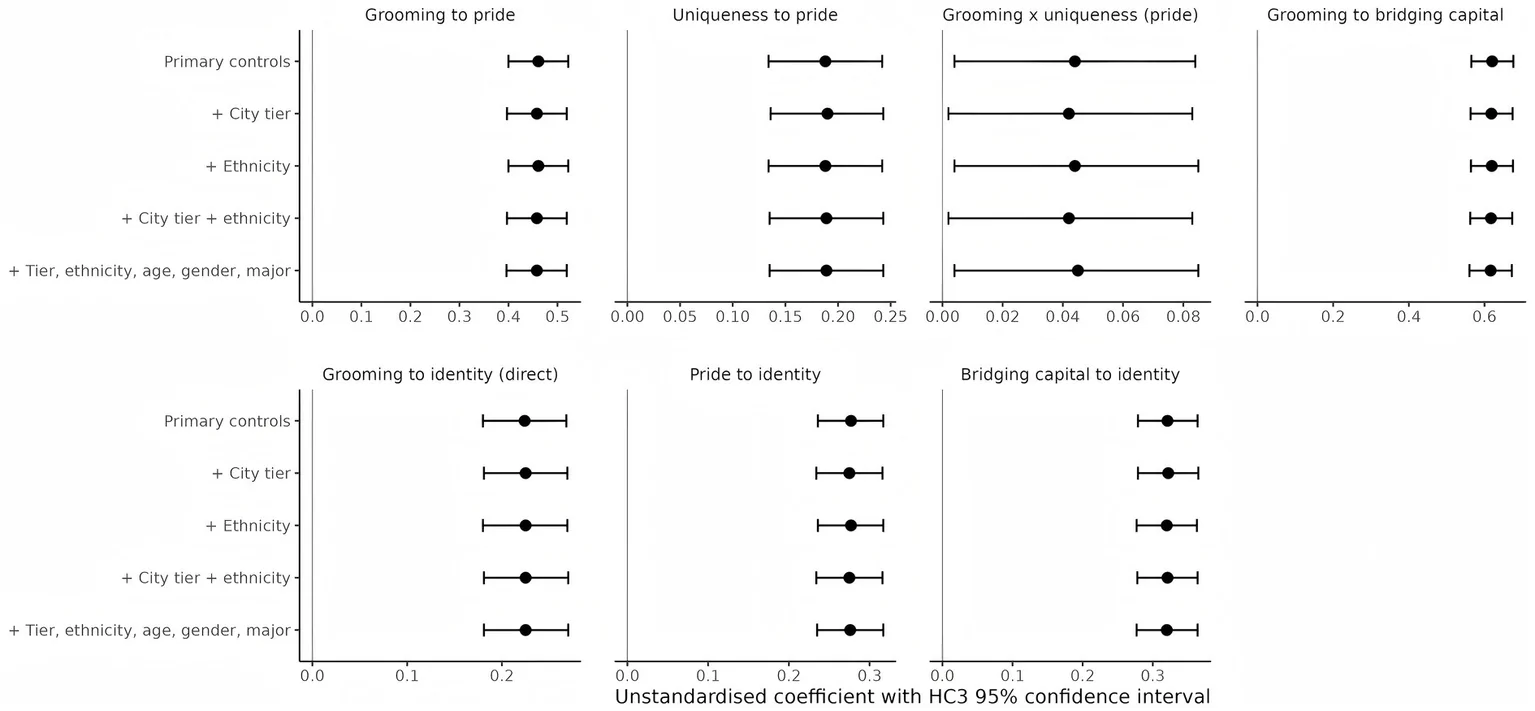
Focal coefficients of the primary model across control specifications. Note. Points are unstandardised coefficients and bars are HC3 95% confidence intervals. Specifications add, to the three primary controls (English proficiency, number of foreign languages studied, daily social media time), city tier of the institution, ethnicity, both, and both together with age, gender and field of study.

A latent-variable structural model reproduced the nonmoderated direct and intervening-variable associations, but it did not contain either interaction and therefore does not validate the moderation findings. Alternative global and dimension-level identity scorings also retained positive direct and indirect associations. These results are reported in Appendix Tables 19, 20.

In language-study subgroups, the HC3 *p* values for the pride interaction were 0.401 among participants who had studied one foreign language and 0.048 among those who had studied two or more. The formal three-way cross-group interaction had HC3 *p* = 0.300, so no group difference was detected (Appendix Table 21). Reverse-order regressions showed that the covariance pattern can equally be represented with identity preceding grooming (Appendix Table 22). Finally, the exploratory negative relational interaction was similar without controls, with extended controls, with dummy-coded ordered controls, and after the longstring sensitivity screen (Appendix Table 23).

English proficiency was examined as a moderator in a supplementary analysis (Appendix Table 40). The grooming–pride association was stronger at higher proficiency (interaction *b* = 0.070, HC3 95% CI [0.024, 0.115], *p* = 0.003, *f*^2^ = 0.014; simple slopes 0.372 one standard deviation below the proficiency mean and 0.548 one standard deviation above), as was the direct grooming–identity association (*b* = 0.033, [0.007, 0.058], *p* = 0.013, *f*^2^ = 0.010; slopes 0.185 and 0.268), whereas the grooming–bridging association did not vary with proficiency (*b* = 0.035, [−0.010, 0.080], *p* = 0.125). All three associations remained positive at low proficiency. Proficiency did not account for the grooming–identity association: the partial correlation controlling for proficiency was 0.716 against a zero-order correlation of 0.718.

## 5 Discussion

Before turning to what the present study observed, it is useful to restate the conceptual problem it was designed to explore. In contemporary theorising, multilingual identity is understood as a self-perception negotiated through participation, investment, and social recognition; it does not follow automatically from linguistic proficiency (Darvin and Norton, 2015; Fisher et al., 2020; Norton, 2013). On social media, such participation is increasingly a matter of visible production: what people post, caption, and reply to, and in which languages, is part of how they present themselves and come to understand who they are (Androutsopoulos, 2015; Goffman, 1959; Marwick and Boyd, 2011). The study approached this visible, relational language use through the lens of social grooming, the everyday communicative work of acknowledging, responding to, and staying connected with others (Dunbar, 2004; Lin, 2019; Tufekci, 2008), and asked how it might be bound up with a multilingual sense of self.

Three further bodies of theory framed the processes of interest. Work on self-conscious emotion suggests that competent, effortful performance can be appraised with authentic pride, an emotion tied to the self and to the consolidation of valued attributes (Fredrickson, 2001; Tracy and Robins, 2007; Williams and DeSteno, 2008). Social-capital theory links communicative reach across heterogeneous, weakly tied contacts with access to novel perspectives and a sense of expanded belonging (Ellison et al., 2007; Granovetter, 1973; Putnam, 2000; Williams, 2006). Uniqueness and optimal-distinctiveness theory holds that the personal significance of a distinctive practice varies with how far the individual values being different (Brewer, 1991; Lynn and Harris, 1997; Snyder and Fromkin, 1977). Bringing these strands together, the study framed multilingual production as potentially associated with multilingual identity through an intrapersonal, affective process and an interpersonal, relational process, with a dispositional preference for distinctiveness proposed as a moderator of the affective process.

The prespecified results were consistent with the proposed framework. Multilingual social grooming was positively associated with self-perceived multilingual identity directly and through two parallel indirect associations. One involved authentic pride and the other online bridging social capital; the relational indirect association was larger. The grooming-pride association and the corresponding conditional indirect association were stronger at higher levels of need for uniqueness. The moderation was small in effect size (*f*^2^ = 0.010), and the grooming–pride association was positive across the observed range.

The exploratory extension added a small negative interaction between grooming and need for uniqueness in the bridging-capital equation (*f*^2^ = 0.007): the positive grooming–bridging association was slightly weaker at higher uniqueness and remained positive at every examined level. The affective and relational interactions therefore had opposite signs, and both were small.

The primary direct and indirect associations were broadly similar across the reported sensitivity analyses. The nonmoderated latent model supported the main structural pattern but could not evaluate moderation. Subgroup estimates did not establish that the affective interaction differed by number of languages studied. None of these analyses resolves temporal order or causation.

The positive association between relational multilingual production and multilingual identity extends work that has concentrated largely on multilingual consumption and learning. It complements evidence linking media consumption with self-perceived multilingual identity (Ren and Wang, 2026) by showing that outward, communicative production is associated with the same self-understanding. This pattern is compatible with accounts of identity as negotiated through investment and participation (Darvin and Norton, 2015; Norton, 2013) and with holistic conceptions of multilingual identity as an orientation towards one’s full repertoire (Fisher et al., 2020; Shi and Xu, 2026). Conceptualising the behaviour as social grooming also connects multilingual production with relationship-maintenance practices (Lin, 2019; Tufekci, 2008; Wang and Wang, 2025).

The affective process is consistent with theory on self-conscious emotion. Authentic pride concerns accomplishment-based self-evaluation (Tracy and Robins, 2007), and its association with multilingual identity is compatible with the idea that positive self-conscious emotion accompanies consolidation of valued attributes (Fredrickson, 2001; Williams and DeSteno, 2008). The present contribution is to locate this association within relational multilingual production and to show that it is slightly stronger among people who place greater value on distinctiveness.

The relational process is consistent with social-capital accounts of weak ties, novel perspectives, and expanded social reach (Ellison et al., 2007; Granovetter, 1973; Putnam, 2000; Williams, 2006). The larger indirect association through bridging capital suggests that perceived communicative reach is closely related to multilingual self-understanding in this sample.

The two interaction directions suggest a more differentiated role for uniqueness than the initial account proposed. A preference for distinctiveness may increase the self-evaluative significance attached to multilingual performance, while slightly reducing how strongly the same behaviour covaries with general bridging capital. One possible interpretation is that distinctive self-expression is especially relevant to self-appraisal but somewhat less tightly coupled to broad relational orientation. This interpretation is an inference, not a measured mechanism. The relational interaction was *post hoc*, small, and based on the same sample, so it requires preregistered replication before it should be incorporated into a stable theoretical model.

English proficiency enters the primary model as a background condition: the model concerns what users do with their languages, multilingual identity is self-perceived (Fisher et al., 2020; Shi and Xu, 2026), and grooming is defined by relational use. The supplementary analysis clarifies the relation between the two. Proficiency did not explain the grooming-identity association, which was unchanged when proficiency was partialled out, so the two are complementary correlates of multilingual self-understanding. Proficiency did, however, condition the affective and direct associations: the grooming-pride association was stronger, and the direct grooming-identity association slightly stronger, among more proficient students, and the relational association did not vary with proficiency. This pattern fits the appraisal account of authentic pride, in which more proficient users have more occasions to read their multilingual communication as competent, effortful accomplishment (Tracy and Robins, 2007), while the relational reach of grooming depends on ties. Like the uniqueness interactions, the proficiency interactions were small in effect size.

First, the study extends identity-oriented language research from media consumption towards media production. It frames visible multilingual communication as relational action. Second, it places affective and relational correlates within one associational model of multilingual identity. Third, the combined prespecified and exploratory results indicate that need for uniqueness may relate differently to the two processes: the affective association was stronger, and the relational association slightly weaker, at higher levels of the disposition. This divergence indicates that emotional and relational correlates are not interchangeable.

Need for uniqueness captures one axis of individual variation, a dispositional preference for distinctiveness. A second axis, largely unmeasured here, is the family and social environment in which multilingual repertoires are formed. Tang and Calafato (2025) found, among 156 Chinese university students, that exposure to multilingual practices at home and belief in the benefits of a multilingual upbringing predicted the multilingual practices students envisaged for their own future families, and that fluid use of the repertoire at university was associated with more deliberate intended language management, which suggests that multilingual experience at university carries forward into the language policies students envision for their own families. Visible multilingual production on social media may form part of that loop, but the present model did not include home-language practices, family language beliefs or ethnic background, and the differentiated role found for uniqueness is one source of individual variation among several.

Educators may find it useful to recognize students’ out-of-class multilingual social media production as a potentially identity-relevant form of communication. Designers and moderators of online communities may likewise consider how multilingual expression supports participation across audiences. The exploratory divergence between the affective and relational processes also suggests that users may not experience multilingual production in a single uniform way.

The design was cross-sectional, and three orderings are consistent with the covariance it records. In the theorised ordering, visible multilingual production is accompanied by affective and relational returns that covary with self-perception. In the reverse ordering, students with a stronger multilingual self-perception communicate more visibly, take more pride in doing so, and perceive greater relational reach; the reverse-order regressions (Appendix Table 22) show the data are equally representable this way. A reciprocal ordering, in which production and self-perception feed each other over time, fits most closely with the identity theory the study draws on, since participation and self-understanding are there mutually constitutive (Darvin and Norton, 2015; Norton, 2013). Cross-lagged and random-intercept panel designs, intensive longitudinal measurement, and experiments that vary communication context or audience response will separate these orderings.

All focal constructs were self-reported on one occasion, and the latent method factor captured a substantial general component. The interaction results are secure against this: common method variance attenuates interaction effects (Siemsen et al., 2010). The linear associations may carry inflation, and the grooming-identity and bridging-identity correlations are large enough for the question to matter. Measuring the outcome on a separate occasion, even two weeks after the predictors, and adding behavioural records of multilingual posting, will put the linear estimates on the same footing as the interactions.

The sample was recruited by a survey company from institutions in first- to fourth-tier cities and included 40 ethnic minority students (5.3%). City tier left every estimate unchanged, the minority and Han subsamples were alike on grooming, pride and identity, and the minority subsample was powered for standardised differences of 0.46 and larger. Tang and Calafato (2024) found, in a latent profile analysis of 221 students in southwest China, that minority students clustered in a profile marked by more varied home languages, proficiency in more languages, more fluid translingual practice and higher intercultural effectiveness; the higher self-rated English proficiency of minority students here (g = 0.36) points in a compatible direction. Family language environment forms a further axis of individual variation beyond the dispositional one modelled here: exposure to multilingual practices at home and beliefs about a multilingual upbringing shape the language practices students envisage for their own families, and university multilingual experience carries forward into envisioned family language management (Tang and Calafato, 2025). Larger and purposively recruited minority samples, with the home-language repertoire and family language beliefs measured directly, will show how far the grooming-identity association holds across ethnic and family backgrounds.

The grooming measure aggregates English-only and mixed Chinese-English communication across posts, comments and replies. The independent sample supports this aggregation, since the three content facets were empirically inseparable and the score was related to yet distinct from overall posting, commenting, browsing, private messaging, and proficiency (Appendix Section 9); the language composition of individual acts remains unrecorded. A design that measures grooming in the societal language and in the additional language in parallel will isolate the specifically multilingual increment of the relational work, and test–retest reliability and criterion-related validity against behavioural records of multilingual posting are the corresponding measurement steps.

The outcome measured the evaluative dimension of multilingual identity; the experience and emotion dimensions of the framework (Fisher et al., 2024; Shi and Xu, 2026) were reserved to keep the outcome distinct from the behavioural predictor and the affective intervening variable. The findings therefore speak to multilingual self-perception, and some discriminant comparisons involving this evaluative outcome sat below the heterotrait-monotrait results, consistent with its adjacency to positive self-appraisal and perceived relational reach. The general online bridging measure covered social media interaction at large, and need for uniqueness is a general disposition. The full three-dimensional identity instrument administered on a separate occasion, a bridging measure specific to multilingual exchanges, and domain-specific dispositional measures will sharpen these tests.

The model contains the positive returns of visible multilingual production, and both interactions were small (*f*-squared at or below 0.01), with the relational interaction exploratory. Visible additional-language use can also be read as display, exclusion or status-seeking, and English holds a distinctive symbolic position in Chinese higher education and digital culture, as a compulsory subject, a gatekeeping credential, and a marker of cosmopolitan orientation, against which such readings form. The next studies should therefore measure audience composition, feedback and negative reception alongside the positive returns, replicate the relational moderation with power adequate for small interactions, and re-operationalise the construct for ecologies where the additional language is a heritage, regional or minority language and for repertoires beyond two languages, so that the affective and relational associations can be compared across settings.

## 6 Conclusion

Multilingual social grooming was positively associated with self-perceived multilingual identity directly and through authentic pride and online bridging social capital. The grooming-pride association and its indirect association with identity were modestly stronger at higher levels of need for uniqueness. An exploratory extension found that the positive grooming–bridging association was slightly weaker at higher levels of uniqueness; both interactions were small in effect size (*f*^2^ < 0.02). The study therefore supports an associational account in which visible multilingual production covaries with both affective self-appraisal and perceived relational reach, and in which a preference for distinctiveness relates differently to the two.

## Data Availability

The raw data supporting the conclusions of this article will be made available by the authors, without undue reservation.

## References

[ref1] Androutsopoulos J. (2015). Networked multilingualism: some language practices on Facebook and their implications. Int. J. Bilingualism 19, 185–205. doi: 10.1177/1367006913489198

[ref2] Block D. (2007). Second Language Identities. London, UK: Continuum.

[ref3] Brewer M. B. (1991). The social self: on being the same and different at the same time. Personal. Soc. Psychol. Bull. 17, 475–482. doi: 10.1177/0146167291175001

[ref4] Chu S.-C. Deng T. Mundel J. (2024). The impact of personalization on viral behavior intentions on TikTok: the role of perceived creativity, authenticity, and need for uniqueness. J. Mark. Commun. 30, 1–20. doi: 10.1080/13527266.2022.2098364

[ref5] Cohen J. (1988). Statistical Power Analysis for the Behavioral Sciences. 2nd Edn. Hillsdale, NJ: Lawrence Erlbaum.

[ref6] Darvin R. Norton B. (2015). Identity and a model of investment in applied linguistics. Annu. Rev. Appl. Linguist. 35, 36–56. doi: 10.1017/S0267190514000191

[ref7] Dörnyei Z. (2009). “The L2 motivational self system,” in Motivation, Language Identity and the L2 Self, eds. Dörnyei Z. Ushioda E. (Bristol, UK: Multilingual Matters), 9–42.

[ref8] Douglas Fir Group (2016). A transdisciplinary framework for SLA in a multilingual world. Mod. Lang. J. 100, 19–47. doi: 10.1111/modl.12301

[ref9] Dunbar R. I. M. (2004). Gossip in evolutionary perspective. Rev. Gen. Psychol. 8, 100–110. doi: 10.1037/1089-2680.8.2.100

[ref10] Dunn A. M. Heggestad E. D. Shanock L. R. Theilgard N. (2018). Intra-individual response variability as an indicator of insufficient effort responding: comparison to other indicators and relationships with individual differences. J. Bus. Psychol. 33, 105–121. doi: 10.1007/s10869-016-9479-0

[ref11] Ebadi S. Zandi M. Fakher Ajabshir Z. (2024). The effect of language learning engagement on L2 motivation over Instagram. Acta Psychol. 250:104512. doi: 10.1016/j.actpsy.2024.104512, 39361988

[ref12] Eg R. Demirkol Tønnesen Ö. Tennfjord M. K. (2023). A scoping review of personalized user experiences on social media: the interplay between algorithms and human factors. Comput. Hum. Behav. Rep. 9:100253. doi: 10.1016/j.chbr.2022.100253, 42574925

[ref13] Ellison N. B. Steinfield C. Lampe C. (2007). The benefits of Facebook “friends”: social capital and college students’ use of online social network sites. J. Comput. Mediat. Commun. 12, 1143–1168. doi: 10.1111/j.1083-6101.2007.00367.x

[ref14] Eren Ö. (2025). Multilingualism in digital spaces: utilising telecollaboration to raise multilingual awareness in language teacher education. Int. J. Multiling. 22, 1340–1358. doi: 10.1080/14790718.2024.2377192

[ref15] Fisher L. Evans M. Forbes K. Gayton A. Liu Y. (2020). Participative multilingual identity construction in the languages classroom: a multi-theoretical conceptualisation. Int. J. Multiling. 17, 448–466. doi: 10.1080/14790718.2018.1524896

[ref1101] Fisher L. Evans M. Forbes K. Gayton A. Liu Y. Rutgers D. (2024). Language experiences, evaluations and emotions (3Es): analysis of structural models of multilingual identity for language learners in schools in England. Int. J. Multiling. 21, 418–438. doi: 10.1080/14790718.2022.2060235

[ref16] Fornell C. Larcker D. F. (1981). Evaluating structural equation models with unobservable variables and measurement error. J. Mark. Res. 18, 39–50. doi: 10.1177/002224378101800104

[ref17] Fredrickson B. L. (2001). The role of positive emotions in positive psychology: the broaden-and-build theory of positive emotions. Am. Psychol. 56, 218–226. doi: 10.1037/0003-066X.56.3.218, 11315248 PMC3122271

[ref18] Giles H. Johnson P. (1987). Ethnolinguistic identity theory: a social psychological approach to language maintenance. Int. J. Sociol. Lang., 1987(68), 69–99. doi:doi: 10.1515/ijsl.1987.68.69

[ref19] Goffman E. (1959). The Presentation of Self in Everyday Life. New York, NY: Doubleday.

[ref20] Granovetter M. S. (1973). The strength of weak ties. Am. J. Sociol. 78, 1360–1380. doi: 10.1086/225469

[ref21] Hayes A. F. (2022). Introduction to Mediation, Moderation, and Conditional Process Analysis: A Regression-based Approach. 3rd Edn. New York, NY: Guilford Press.

[ref22] Helmond A. (2026). The platformization of everything: from the end of the like button to AI infrastructure in space. Soc. Media + Soc. 12. doi: 10.1177/20563051261436101

[ref23] Henseler J. Ringle C. M. Sarstedt M. (2015). A new criterion for assessing discriminant validity in variance-based structural equation modeling. J. Acad. Mark. Sci. 43, 115–135. doi: 10.1007/s11747-014-0403-8

[ref24] Hu L. Bentler P. M. (1999). Cutoff criteria for fit indexes in covariance structure analysis: conventional criteria versus new alternatives. Struct. Equ. Model. 6, 1–55. doi: 10.1080/10705519909540118

[ref25] Khajavy G. H. Lüftenegger M. (2025). Pride in foreign language learning: a conceptual framework and empirical evidence. Innov. Lang. Learn. Teach. 19, 379–394. doi: 10.1080/17501229.2024.2361652

[ref26] Li W. (2018). Translanguaging as a practical theory of language. Appl. Linguist. 39, 9–30. doi: 10.1093/applin/amx039

[ref27] Lin J.-H. T. (2019). Strategic social grooming: emergent social grooming styles on Facebook, social capital and well-being. J. Comput. Mediat. Commun. 24, 90–107. doi: 10.1093/jcmc/zmz002

[ref28] Liu P. L. Yeo T. E. D. (2024). Social grooming on social media and older adults’ life satisfaction: testing a moderated mediation model. Soc. Sci. Comput. Rev. 42, 913–929. doi: 10.1177/08944393231220487

[ref29] Lynn M. Harris J. (1997). Individual differences in the pursuit of self-uniqueness through consumption. J. Appl. Soc. Psychol. 27, 1861–1883. doi: 10.1111/j.1559-1816.1997.tb01629.x

[ref30] Marwick A. E. Boyd D. (2011). I tweet honestly, I tweet passionately: twitter users, context collapse, and the imagined audience. New Media Soc. 13, 114–133. doi: 10.1177/1461444810365313

[ref31] Meade A. W. Craig S. B. (2012). Identifying careless responses in survey data. Psychol. Methods 17, 437–455. doi: 10.1037/a0028085, 22506584

[ref32] Narayan D. (2024). The political economy of digital platforms: key directions. Platforms Soc. 1. doi: 10.1177/29768624241263071

[ref33] Norton B. (2013). Identity and Language Learning: Extending the Conversation. 2nd Edn Bristol, UK: Multilingual Matters.

[ref34] Norton Peirce B. (1995). Social identity, investment, and language learning. TESOL Q. 29, 9–31. doi: 10.2307/3587803

[ref35] Podsakoff P. M. MacKenzie S. B. Lee J.-Y. Podsakoff N. P. (2003). Common method biases in behavioral research: a critical review of the literature and recommended remedies. J. Appl. Psychol. 88, 879–903. doi: 10.1037/0021-9010.88.5.879, 14516251

[ref36] Putnam R. D. (2000). Bowling Alone: The Collapse and Revival of American Community. New York, NY: Simon & Schuster.

[ref37] Ren Y. Wang X. (2026). Media consumption and self-perceived multilingual identity in short-video vlog-assisted informal digital language learning. Front. Psychol. 17. doi: 10.3389/fpsyg.2026.1915495, 42528960 PMC13416255

[ref38] Shi J. Xu J. (2026). Assessing multilingual identity of Chinese university students: scale development and validation. J. Multiling. Multicult. Dev. 47, 4503–4528. doi: 10.1080/01434632.2025.2555529

[ref39] Siemsen E. Roth A. Oliveira P. (2010). Common method bias in regression models with linear, quadratic, and interaction effects. Organ. Res. Methods 13, 456–476. doi: 10.1177/1094428109351241

[ref40] Snyder C. R. Fromkin H. L. (1977). Abnormality as a positive characteristic: the development and validation of a scale measuring need for uniqueness. J. Abnorm. Psychol. 86, 518–527. doi: 10.1037/0021-843X.86.5.518

[ref41] Sulis G. Mairitsch A. Mercer S. (2025). The more languages you know, the better you are’: learners’ pride in being multilingual and their motivation for language learning. J. Multiling. Multicult. Dev. doi: 10.1080/01434632.2025.2576616

[ref42] Tajfel H. (1974). Social identity and intergroup behaviour. Soc. Sci. Inf. 13, 65–93. doi: 10.1177/053901847401300204

[ref43] Tajfel H. Turner J. C. (1986). “The social identity theory of intergroup behavior,” in Psychology of Intergroup Relations, eds. Worchel S. Austin W. G.. 2nd ed (Chicago, IL: Nelson-Hall), 7–24.

[ref44] Tang F. Calafato R. (2024). Exploring plurilingualism, ethnicity, and intercultural effectiveness: insights from university students. Eur. J. Educ. 59:e12738. doi: 10.1111/ejed.12738

[ref45] Tang F. Calafato R. (2025). Family language policy: the impact of multilingual experiences at university and language practices at home. Int. J. Biling. Educ. Biling. 28, 92–107. doi: 10.1080/13670050.2024.2409114

[ref46] Taylor S. H. Choi M. (2022). An initial conceptualization of algorithm responsiveness: comparing perceptions of algorithms across social media platforms. Soc. Media + Soc. 8. doi: 10.1177/20563051221144322

[ref47] Tracy J. L. Robins R. W. (2004). Putting the self into self-conscious emotions: a theoretical model. Psychol. Inq. 15, 103–125. doi: 10.1207/s15327965pli1502_01

[ref48] Tracy J. L. Robins R. W. (2007). The psychological structure of pride: a tale of two facets. J. Pers. Soc. Psychol. 92, 506–525. doi: 10.1037/0022-3514.92.3.506, 17352606

[ref49] Tuck A. B. Thompson R. J. (2024). The social media use scale: development and validation. Assessment 31, 617–636. doi: 10.1177/10731911231173080, 37226810

[ref50] Tufekci Z. (2008). Grooming, gossip, Facebook and Myspace: what can we learn about these sites from those who won’t assimilate? Inf. Commun. Soc. 11, 544–564. doi: 10.1080/13691180801999050

[ref51] van Dijck J. (2013). The Culture of Connectivity: A Critical History of Social Media. New York, NY: Oxford University Press.

[ref52] Wang F. Wang X. (2025). Let’s post more! The impact of foreign language social grooming on social media on learners’ enjoyment: a moderated mediation model. Front. Psychol. 16:1674786. doi: 10.3389/fpsyg.2025.1674786, 41132601 PMC12540393

[ref53] Williams D. (2006). On and off the ‘net: scales for social capital in an online era. J. Comput.-Mediat. Commun. 11, 593–628. doi: 10.1111/j.1083-6101.2006.00029.x

[ref54] Williams L. A. DeSteno D. (2008). Pride and perseverance: the motivational role of pride. J. Pers. Soc. Psychol. 94, 1007–1017. doi: 10.1037/0022-3514.94.6.1007, 18505314

[ref55] Zhang X. Li J. Gu Z. Gao X. (2026). How does language learning contribute to individual growth in a multilingual world? A systematic review. J. Multiling. Multicult. Dev. 47, 4630–4646. doi: 10.1080/01434632.2025.2562096

[ref56] Zou M. M. Liu G. L. Li D. Chen H. (2025). Beyond motivation: modeling the predictive role of L2 pride in informal digital learning of English. Int. J. Appl. Linguist. 35, 1938–1952. doi: 10.1111/ijal.12738

