# Supplementary material for: Multilingual social grooming on social media and self-perceived multilingual identity among Chinese university students
Source: Front Psychol. 2026 Sep 23;17:1942436. doi: 10.3389/fpsyg.2026.1942436 (PMC13642788; doi:10.3389/fpsyg.2026.1942436)
Supplement: Supplementary file 1 [file Data_Sheet_1.PDF]

Supplementary Material

These appendices report supporting descriptive, measurement, diagnostic, exploratory, and sensitivity analyses for the 755 retained cases. Construct and statistical names are written in full where space permits. All analyses are associational.

1. Sample composition and item-level statistics

1.1 Participant and control-variable distributions

Appendix Table 1 reports the complete demographic and control-variable distribution.

Appendix Table 1

Participant and control-variable distributions.

Characteristic	Number and percentage, or mean and standard deviation
Gender: men	320 (42.4%)
Gender: women	435 (57.6%)
Age in years	20.00 (1.60), range 18-24
Field: humanities and social sciences	265 (35.1%)
Field: science, technology, engineering, and mathematics	208 (27.5%)
Field: business and economics	184 (24.4%)
Field: arts and sports	98 (13.0%)
Foreign languages studied: one, English only	374 (49.5%)
Foreign languages studied: two	286 (37.9%)
Foreign languages studied: three or more	95 (12.6%)
Self-rated English proficiency	4.15 (1.26)
Daily social media time: less than 30 minutes	71 (9.4%)
Daily social media time: 30-59 minutes	168 (22.3%)
Daily social media time: 1-2 hours	303 (40.1%)
Daily social media time: more than 2 and up to 3 hours	134 (17.7%)
Daily social media time: more than 3 hours	79 (10.5%)

Note. N = 755. Values for age and English proficiency are means with standard deviations in parentheses.

1.2 Item-level descriptive statistics

The following tables replace item codes with the complete English wording of each question. They therefore provide both the item-level descriptive analysis and the wording needed to interpret it, without a separate duplicated questionnaire.

1.2.1 Multilingual social grooming

Appendix Table 2 reports item-level results for multilingual social grooming.

Appendix Table 2

Multilingual social grooming: complete item wording and item-level statistics.

Question wording	Mean	Standard deviation	Skewness	Kurtosis	Corrected item-total correlation	Standardised loading
I use English or Chinese-English mixed captions or posts to share my daily experiences with other people on social media.	4.61	1.61	-.34	-.46	.73	.76
I use English or Chinese-English mixed posts or captions to express my thoughts and feelings to other people on social media.	4.78	1.68	-.45	-.51	.75	.79
I use English or Chinese-English mixed expressions to acknowledge, encourage, or support other people on social media.	4.62	1.63	-.36	-.53	.78	.82
I use English or Chinese-English mixed comments to respond to other people's posts on social media.	4.79	1.68	-.40	-.65	.78	.81
I reply in English or in mixed Chinese-English to comments that other people leave on my posts.	4.36	1.52	-.24	-.43	.76	.79
I use English or Chinese-English mixed greetings, wishes, or brief supportive messages to interact with other people on social media.	4.67	1.63	-.38	-.50	.76	.80
I use English or Chinese-English mixed communication to keep in touch with people I do not often see in person.	4.73	1.64	-.39	-.54	.74	.77
I use English or Chinese-English mixed communication to maintain my relationships with other people on social media.	4.55	1.57	-.33	-.40	.70	.73

Note. N = 755. Response scale: 1 = never; 2 = less than once a month; 3 = about once a month; 4 = several times a month; 5 = about once a week; 6 = several times a week; 7 = daily or almost daily. The reference period was the previous three months. Standardised loadings are from the distinct-construct confirmatory model.

1.2.2 Authentic pride

Appendix Table 3 reports item-level results for authentic pride.

Appendix Table 3

Authentic pride: complete item wording and item-level statistics.

Question wording	Mean	Standard deviation	Skewness	Kurtosis	Corrected item-total correlation	Standardised loading
I feel accomplished.	4.37	1.52	-.19	-.43	.71	.75
I feel like I am achieving.	4.81	1.63	-.44	-.47	.69	.72
I feel confident.	4.47	1.56	-.25	-.46	.73	.77
I feel fulfilled.	4.54	1.57	-.28	-.45	.73	.77
I feel productive.	4.82	1.62	-.49	-.46	.77	.82
I feel a sense of self-worth.	4.39	1.55	-.24	-.46	.69	.73
I feel successful.	4.67	1.63	-.41	-.45	.70	.74

Note. N = 755. Response scale: 1 = not at all to 7 = extremely. Participants considered English-only or mixed Chinese-English social media communication during the previous three months. Standardised loadings are from the distinct-construct confirmatory model.

1.2.3 Online bridging social capital

Appendix Table 4 reports item-level results for online bridging social capital.

Appendix Table 4

Online bridging social capital: complete item wording and item-level statistics.

Question wording	Mean	Standard deviation	Skewness	Kurtosis	Corrected item-total correlation	Standardised loading
Interacting with people through social media makes me interested in things that happen outside of my town.	4.52	1.59	-.28	-.47	.77	.80
Interacting with people through social media makes me want to try new things.	4.48	1.58	-.29	-.48	.77	.80
Interacting with people through social media makes me interested in what people unlike me are thinking.	4.92	1.66	-.47	-.52	.75	.78
Talking with people through social media makes me curious about other places in the world.	4.42	1.54	-.22	-.39	.71	.74
Interacting with people through social media makes me feel like part of a larger community.	4.68	1.63	-.39	-.56	.76	.79
Interacting with people through social media makes me feel connected to the bigger picture.	4.36	1.54	-.23	-.38	.74	.77
Interacting with people through social media reminds me that everyone in the world is connected.	4.32	1.53	-.21	-.42	.74	.77
Interacting with people through social media gives me new people to talk to.	4.89	1.68	-.55	-.42	.72	.75
Through social media, I come in contact with new people all the time.	4.76	1.63	-.47	-.39	.77	.80

Note. N = 755. Response scale: 1 = strongly disagree to 7 = strongly agree. Standardised loadings are from the distinct-construct confirmatory model.

1.2.4 Need for uniqueness

Appendix Table 5 reports item-level results for need for uniqueness.

Appendix Table 5

Need for uniqueness: complete item wording and item-level statistics.

Question wording	Mean	Standard deviation	Skewness	Kurtosis	Corrected item-total correlation	Standardised loading
I prefer being different from other people.	4.56	1.62	-.28	-.56	.77	.82
Being distinctive is important to me.	4.39	1.58	-.18	-.49	.78	.84
I intentionally do things to make myself different from those around me.	4.36	1.56	-.22	-.45	.73	.78
I have a need for uniqueness.	4.36	1.55	-.23	-.48	.79	.86

Note. N = 755. Response scale: 1 = strongly disagree to 7 = strongly agree. Standardised loadings are from the distinct-construct confirmatory model.

1.2.5 Self-perceived multilingual identity

Appendix Table 6 reports item-level results for self-perceived multilingual identity.

Appendix Table 6

Self-perceived multilingual identity: complete item wording and item-level statistics.

Question wording	Mean	Standard deviation	Skewness	Kurtosis	Corrected item-total correlation
I can imagine myself using multiple foreign languages in my future job.	4.30	1.52	-.12	-.50	.65
I can imagine myself mastering multiple foreign languages in the future.	4.31	1.54	-.20	-.40	.69
Speaking multiple foreign languages is important to me.	4.63	1.62	-.32	-.53	.68
Whether or not it is required by the school curriculum, I still want to learn another foreign language.	4.41	1.55	-.21	-.48	.71
I see the ability to speak multiple languages as part of my identity.	4.34	1.54	-.17	-.42	.66
I want to become someone who can speak multiple foreign languages fluently in the future.	4.38	1.55	-.20	-.40	.66
The more languages we master, the more creative we become.	4.52	1.60	-.31	-.48	.73
Mastering a new language helps us better understand the languages we have already learned.	4.42	1.56	-.22	-.46	.66
Mastering multiple languages helps us view problems from different perspectives.	4.80	1.62	-.44	-.47	.69
Mastering multiple languages helps us acquire knowledge in other academic subjects more effectively.	4.37	1.53	-.18	-.42	.69
The more languages we master, the easier it is to learn a new language.	4.95	1.64	-.48	-.51	.67
Learning multiple languages changes the way we think.	4.73	1.62	-.39	-.53	.62
If I were to travel abroad, I would like to be able to communicate with local people in their own language.	4.60	1.61	-.34	-.49	.62
When talking to foreigners, using their native language shows respect.	4.70	1.62	-.41	-.52	.64
I believe that our Chinese culture and foreign cultures can coexist harmoniously.	4.28	1.54	-.19	-.41	.63
I think that learning an additional foreign language helps me better understand my own culture.	4.47	1.59	-.27	-.46	.64

Note. N = 755. Response scale: 1 = strongly disagree to 7 = strongly agree. The first six items represent evaluation of self, the next six represent evaluation of language, and the final four represent evaluation of culture. Item loadings from a flat single-factor identity model are not reported because the higher-order three-dimensional model is the primary identity specification.

1.3 Composite descriptive statistics

Appendix Table 7 reports the distributional statistics for all composite scores.

Appendix Table 7

Composite descriptive statistics.

Construct	Mean	Standard deviation	Minimum	Maximum	Skewness	Kurtosis
Multilingual social grooming	4.64	1.32	1.00	7.00	-.34	-.31
Authentic pride	4.58	1.26	1.00	7.00	-.32	-.14
Online bridging social capital	4.59	1.29	1.00	7.00	-.46	-.02
Need for uniqueness	4.42	1.37	1.00	7.00	-.24	-.33
Self-perceived multilingual identity	4.51	1.12	1.06	7.00	-.26	.23

Note. N = 755. All composite scores are item means on seven-point metrics.

1.4 Eligibility, attention-check, and demographic item wording

The following items complete the questionnaire record. Together with the focal item wording reproduced in Appendix Tables 2 through 6, they constitute the full instrument. The survey was administered in Chinese; the English wording is reproduced here.

Eligibility screening items (yes / no; respondents answering no to any item did not continue): (1) Are you at least 18 years old? (2) Are you currently enrolled at a college or university in mainland China? (3) Do you use at least one social media platform regularly? (4) During the past three months, have you used English, or a mixture of Chinese and English, at least once in a social media post, caption, comment, or public or semi-public reply?

Instructed-response attention check (embedded within the agreement-scale blocks): “To show that you are reading carefully, please select ‘disagree’ (2) for this item.”

Demographic and control questions: (1) What is your gender? (man / woman) (2) What is your age in years? (open numeric response) (3) Which of the following best describes your field of study? (humanities and social sciences / science, technology, engineering, and mathematics / business and economics / arts and sports) (4) How would you rate your overall English proficiency? (1 = very low to 7 = very high) (5) How many foreign languages have you studied systematically, currently or in the past? Please count English; do not count Chinese or Chinese regional varieties. (one — English only / two / three or more) (6) On average, how much time do you spend on social media per day? (less than 30 minutes / 30–59 minutes / 1–2 hours / more than 2 and up to 3 hours / more than 3 hours)

2. Measurement evidence

2.1 Higher-order identity model

Appendix Table 8 reports the confirmatory evidence used to represent identity as a higher-order construct.

Appendix Table 8

Confirmatory evidence for the three-dimensional and higher-order identity specifications.

Model or loading	Chi-square or loading	Degrees of freedom	Comparative fit index	Tucker-Lewis index	Root-mean-square error	Standardised residual
Correlated three-factor identity model	113.76	101	.998	.998	.013	.021
Five-construct model with higher-order identity	1109.25	889	.990	.989	.018	Not available
Second-order loading: envisioned multilingual self and identity centrality	.84					
Second-order loading: envisioned linguistic and cognitive advantages	.81					
Second-order loading: envisioned intercultural understanding and orientation	.84					

Note. N = 755. The higher-order specification is the primary measurement representation used to justify a global identity score.

2.2 Reliability and convergent validity

Appendix Table 9 separates first-order reliability and convergent-validity evidence from the internal consistency of the higher-order identity dimensions.

Appendix Table 9

Reliability and convergent-validity evidence.

Construct or dimension	Items	Cronbach's alpha	Composite reliability	Average variance extracted
Multilingual social grooming	8	.927	.927	.615
Authentic pride	7	.905	.905	.577
Online bridging social capital	9	.932	.933	.606
Need for uniqueness	4	.895	.895	.681
Self-perceived multilingual identity, global	16	.935		
Envisioned multilingual self and identity centrality	6	.903		
Envisioned linguistic and cognitive advantages	6	.902		
Envisioned intercultural understanding and orientation	4	.861		

Note. Blank cells indicate that a flat first-order composite-reliability or average-variance estimate was not used for the higher-order identity interpretation.

2.3 Discriminant validity

Appendix Table 10 reports heterotrait-monotrait ratios. Appendix Table 11 reports the Fornell-Larcker comparison as a secondary diagnostic.

Appendix Table 10

Heterotrait-monotrait ratios of correlations.

Construct	1	2	3	4	5
Multilingual social grooming					
Authentic pride	.61				
Online bridging social capital	.70	.54			
Need for uniqueness	.36	.39	.34		
Self-perceived multilingual identity	.77	.75	.78	.41	

Note. 1 = multilingual social grooming; 2 = authentic pride; 3 = online bridging social capital; 4 = need for uniqueness; 5 = self-perceived multilingual identity. All ratios were below .85.

Appendix Table 11

Fornell-Larcker matrix.

Construct	1	2	3	4	5
Multilingual social grooming	.78				
Authentic pride	.61	.76			
Online bridging social capital	.70	.54	.78		
Need for uniqueness	.36	.39	.34	.82	
Self-perceived multilingual identity	.77	.75	.78	.41	.69

Note. Diagonal entries are square roots of average variance extracted from the flat first-order diagnostic model; off-diagonal entries are latent correlations. The criterion was not met for several comparisons involving the broad identity construct.

2.4 Alternative measurement models

Appendix Table 12 compares the distinct-construct diagnostic model with less differentiated alternatives. The higher-order model in Appendix Table 8 remains the primary identity specification.

Appendix Table 12

Alternative confirmatory measurement models.

Model	Chi-square	Degrees of freedom	Comparative fit index	Tucker-Lewis index	Root-mean-square error	Standardised residual
Distinct five-factor diagnostic model	2517.91	892	.925	.921	.049	.042
Four-factor model combining grooming and bridging capital	4017.10	896	.857	.849	.068	.055
Four-factor model combining pride and identity	3507.69	896	.880	.874	.062	.054
One-factor model	7711.14	902	.688	.673	.100	.085

Note. N = 755. The flat five-factor model is reported as a discriminant-validity diagnostic, not as the preferred representation of the multidimensional identity construct.

3. Common method variance

3.1 Diagnostic results

Appendix Table 13 reports all three common-method diagnostics cited in the main manuscript.

Appendix Table 13

Common-method diagnostics.

Diagnostic	Index	Result	Interpretation
Harman first unrotated factor	Variance accounted for	41.9%	Below 50%, but not evidence that method variance is absent
One-factor confirmatory model	Comparative fit index; root-mean-square error	.688; .100	Poor fit; covariance is not adequately unidimensional
Unmeasured latent method-factor model	Comparative fit index; root-mean-square error	.964; .035	Improved fit
Unmeasured latent method-factor model	Mean squared method loading	.384	Substantial general component that may mix method and substantive covariance

Note. These diagnostics cannot prove the absence of common method variance.

4. Full correlation matrix

4.1 Focal and control variables

Appendix Table 14 reports zero-order correlations among all focal and control variables.

Appendix Table 14

Zero-order correlations among focal and control variables.

Variable	1	2	3	4	5	6	7	8
1. Multilingual social grooming								
2. Authentic pride	.56***							
3. Online bridging social capital	.65***	.50***						
4. Need for uniqueness	.33***	.35***	.31***					
5. Self-perceived multilingual identity	.72***	.69***	.73***	.37***				
6. Self-rated English proficiency	.18***	.23***	.14***	.03	.39***			
7. Number of foreign languages studied	-.04	-.03	.00	.03	-.00	.06		
8. Daily social media time	.09*	.03	.16***	.11**	.10**	-.05	.04	

Note. N = 755. p < .05; ** p < .01; *** p < .001.*

5. Regression equations, diagnostics, and exploratory extension

5.1 Primary equations

Appendix Table 15 reports all predictors in the three primary equations using HC3 inference.

Appendix Table 15

Complete primary regression equations.

Criterion	Predictor	Coefficient	HC3 standard error	Test statistic	p value
Authentic pride	Intercept	4.17	.19	22.35	< .001
Authentic pride	Multilingual social grooming, mean-centred	.46	.03	14.89	< .001
Authentic pride	Need for uniqueness, mean-centred	.19	.03	6.85	< .001
Authentic pride	Grooming by uniqueness	.04	.02	2.16	.031
Authentic pride	Self-rated English proficiency	.13	.03	4.17	< .001
Authentic pride	Number of foreign languages studied	-.03	.05	-.58	.565
Authentic pride	Daily social media time	-.03	.03	-.95	.344
Online bridging social capital	Intercept	4.03	.19	21.23	< .001
Online bridging social capital	Multilingual social grooming, mean-centred	.62	.03	22.01	< .001
Online bridging social capital	Self-rated English proficiency	.03	.03	1.00	.320
Online bridging social capital	Number of foreign languages studied	.04	.05	.89	.371
Online bridging social capital	Daily social media time	.12	.03	3.70	< .001
Self-perceived multilingual identity	Intercept	.86	.15	5.72	< .001
Self-perceived multilingual identity	Multilingual social grooming, mean-centred	.22	.02	9.98	< .001
Self-perceived multilingual identity	Authentic pride	.28	.02	13.35	< .001
Self-perceived multilingual identity	Online bridging social capital	.32	.02	14.81	< .001
Self-perceived multilingual identity	Self-rated English proficiency	.20	.02	11.53	< .001
Self-perceived multilingual identity	Number of foreign languages studied	.01	.03	.21	.832
Self-perceived multilingual identity	Daily social media time	.02	.02	1.19	.234

Note. Model summaries: authentic pride R-squared = .369; online bridging social capital R-squared = .432; identity R-squared = .762.

5.2 Regression diagnostics

Appendix Table 16 reports the diagnostics that motivated the use of HC3 inference.

Appendix Table 16

Regression diagnostics.

Criterion	Largest variance-inflation factor	Breusch-Pagan p value	Largest Cook's distance	Cases with absolute standardised residual above 3
Authentic pride	1.18	< .001	.030	1
Online bridging social capital	1.05	.025	.028	3
Self-perceived multilingual identity	2.00	.008	.021	2

Note. Breusch-Pagan tests indicated heteroscedasticity in all three equations, so HC3 inference is primary.

5.3 Exploratory relational moderation

Appendix Table 17 reports the post hoc expanded bridging-capital equation and conditional quantities. Appendix Figure 1 displays the three simple slopes.

Appendix Table 17

Exploratory grooming-by-uniqueness moderation of online bridging social capital.

Predictor	Coefficient	HC3 standard error	Test statistic	p value	95% confidence interval
Multilingual social grooming, mean-centred	.58	.03	20.39	< .001	[.53, .64]
Need for uniqueness, mean-centred	.08	.03	2.98	.003	[.03, .14]
Grooming by uniqueness	-.04	.01	-2.55	.011	[-.07, -.01]
Self-rated English proficiency	.04	.03	1.29	.199	[-.02, .10]
Number of foreign languages studied	.03	.05	.68	.498	[-.06, .13]
Daily social media time	.12	.03	3.55	< .001	[.05, .18]

Note. N = 755. R-squared = .444; incremental R-squared for the interaction = .004. The interaction was not part of H1 to H5.

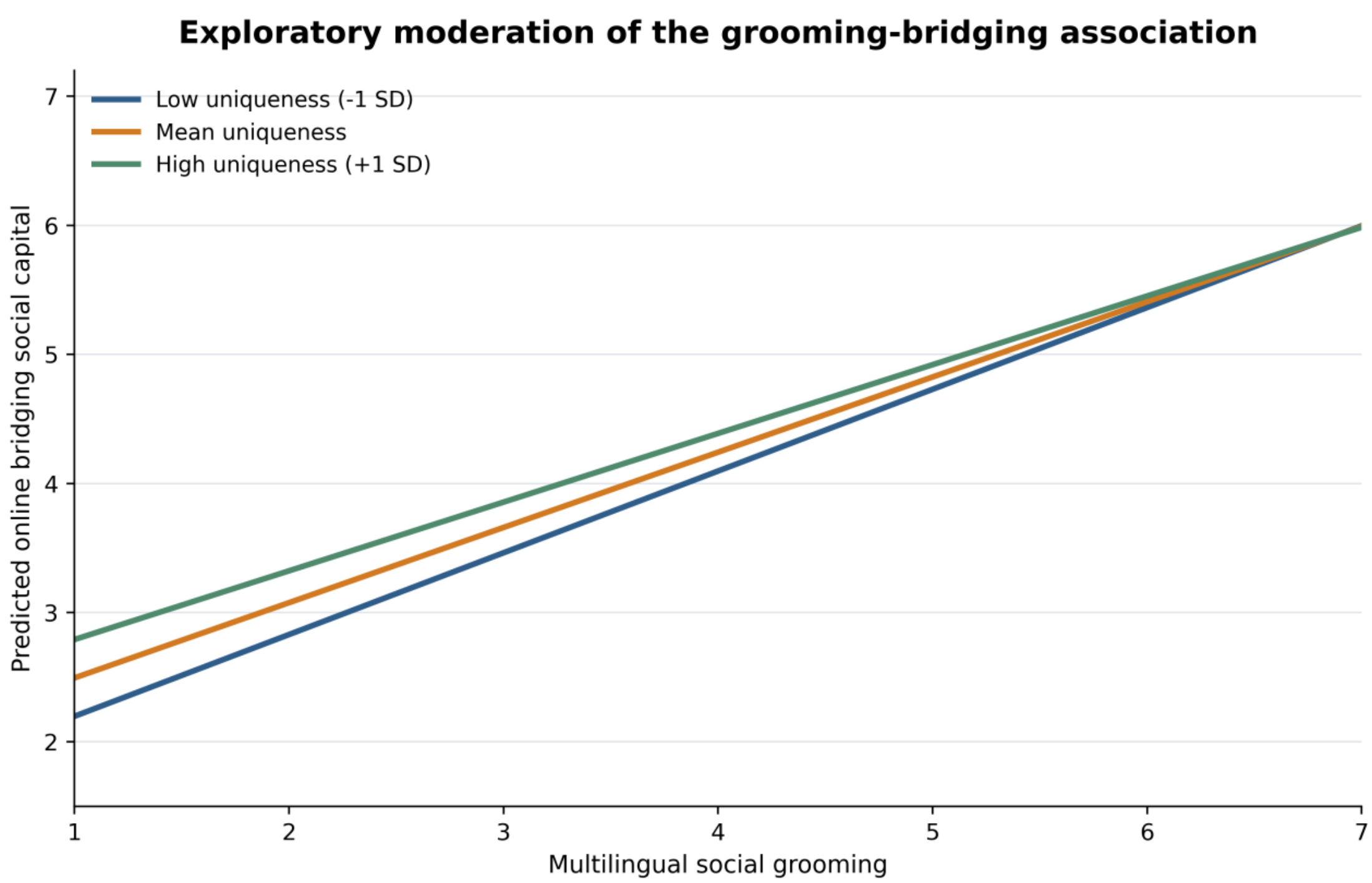

Appendix Figure 1. Exploratory simple slopes of multilingual social grooming on online bridging social capital at low, mean, and high need for uniqueness.

6. Robustness and sensitivity analyses

6.1 Primary composite-score specifications

Appendix Table 18 compares the primary point estimates with three sensitivity specifications.

Appendix Table 18

Primary and sensitivity specifications.

Specification	Grooming to pride	Pride interaction	Grooming to bridging capital	Direct grooming to identity	Indirect through pride	Indirect through bridging capital	Moderated indirect index through pride
Primary controls	.461	.044	.620	.224	.128 [.103, .153]	.199 [.169, .232]	.012 [.002, .024]
No controls	.485	.050	.633	.239	.151	.206	.016
Extended controls	.461	.047	.619	.224	.128 [.104, .154]	.198 [.166, .231]	.013 [.003, .025]
Maximum longstring below 16, n = 744	.436	.056	.594	.217	.115 [.090, .140]	.186 [.154, .217]	.015 [.004, .028]

Note. Extended controls added age, gender, and academic major. The longstring analysis was a sensitivity screen, not part of the primary exclusion rule. The HC3 p values for the pride interaction were .031 in the primary model, .012 without controls, .022 with extended controls, and .017 after the longstring screen.

6.2 Nonmoderated latent-variable structural model

Appendix Table 19 reports the latent-variable robustness model. It did not include moderation and therefore supports only the nonmoderated structural pattern.

Appendix Table 19

Nonmoderated latent-variable structural associations.

Structural association	Standardised coefficient
Multilingual social grooming to authentic pride	.55
Multilingual social grooming to online bridging social capital	.71
Multilingual social grooming to self-perceived multilingual identity	.27
Authentic pride to self-perceived multilingual identity	.39
Online bridging social capital to self-perceived multilingual identity	.39

Note. Model fit: chi-square(895) = 2547.73, comparative fit index = .924, Tucker-Lewis index = .920, root-mean-square error of approximation = .050, standardised root-mean-square residual = .047. Moderation was not estimated.

6.3 Alternative identity scoring

Appendix Table 20 reports the model under a dimension-balanced global score and under each identity dimension separately.

Appendix Table 20

Alternative scoring of self-perceived multilingual identity.

Criterion scoring	Direct grooming	Indirect through authentic	Indirect through online bridging

	coefficient	pride	social capital
Dimension-balanced global score	.23	.13 [.10, .15]	.20 [.17, .23]
Envisioned multilingual self and identity centrality	.25	.13 [.10, .16]	.18 [.14, .23]
Envisioned linguistic and cognitive advantages	.17	.13 [.10, .17]	.21 [.16, .25]
Envisioned intercultural understanding and orientation	.26	.12 [.09, .15]	.21 [.17, .26]

Note. Bracketed values are 95% percentile bootstrap confidence intervals.

6.4 Language-study subgroup analysis

Appendix Table 21 reports subgroup estimates and the formal cross-group test.

Appendix Table 21

Subgroup estimates by number of foreign languages studied.

Quantity	One foreign language	Two or more foreign languages
Number of participants	374	381
Grooming to authentic pride	.485	.435
Grooming by uniqueness interaction	.023	.065
HC3 p value of interaction	.401	.048
Grooming to online bridging social capital	.638	.602
Direct grooming to identity	.249	.200
Indirect through authentic pride	.127	.128
Indirect through online bridging social capital	.191	.207

Note. The formal grooming-by-uniqueness-by-language-group interaction had coefficient .044 and HC3 $p = .300$. The subgroup results do not establish a between-group difference.

6.5 Reverse-order regressions

Appendix Table 22 reports the reverse-order illustration used to demonstrate directional ambiguity.

Appendix Table 22

Selected reverse-order regression estimates.

Association	Coefficient	Standard error	p value
Identity to grooming, controlling for pride, bridging capital, and controls	.568	.055	< .001
Authentic pride to grooming	.122	.037	.001
Online bridging social capital to grooming	.255	.042	< .001
Identity to authentic pride in accompanying equation	.808	.032	< .001
Identity to online bridging social capital in accompanying equation	.900	.031	< .001

Note. The first three rows use HC3 standard errors where available; accompanying equations are

reported to illustrate an alternative ordering. These regressions are not formal comparative model-fit tests and do not establish temporal direction.

6.6 Sensitivity of the exploratory relational interaction

Appendix Table 23 reports the negative relational interaction under alternative specifications.

Appendix Table 23

Sensitivity of the exploratory grooming-by-uniqueness interaction, with online bridging social capital as the criterion variable.

Specification	Number analysed	Interaction coefficient	HC3 standard error	p value	95% confidence interval
No controls	755	-.03	.01	.019	[-.06, -.01]
Primary linear controls	755	-.04	.01	.011	[-.07, -.01]
Dummy-coded ordered controls	755	-.04	.01	.012	[-.07, -.01]
Extended controls	755	-.04	.01	.011	[-.07, -.01]
Maximum longstring below 16	744	-.04	.02	.011	[-.07, -.01]

Note. This post hoc interaction was small and negative in every reported specification.

7. Exploratory factor analysis

7.1 Sampling adequacy and parallel analysis

Sampling adequacy for the 44 focal items was excellent: the overall Kaiser-Meyer-Olkin statistic was .976 (lowest item-level value = .901), and Bartlett's test of sphericity was significant, chi-square(946) = 22,259.5, $p < .001$. Parallel analysis compared each observed eigenvalue with the 95th percentile of eigenvalues from 1,000 random datasets of the same dimensions (Appendix Table 24); six observed eigenvalues exceeded their random-data counterparts, a pattern consistent with the three-dimensional structure of the self-perceived multilingual identity scale.

Appendix Table 24

Observed eigenvalues and parallel-analysis criterion values.

Component	Observed eigenvalue	95th percentile of random-data eigenvalues
1	18.44	1.54
2	2.78	1.48
3	2.38	1.44
4	1.97	1.40
5	1.56	1.37
6	1.47	1.34
7	1.16	1.31
8	.62	1.28

Note. Components whose observed eigenvalue exceeds the random-data criterion are retained under parallel analysis; six components met this criterion.

7.2 Rotated five-factor loadings

The theoretically specified five-factor solution was extracted with principal axis factoring and rotated with the varimax criterion (Appendix Table 25). Thirty-four of the 44 items showed primary loadings of at least .40 on their intended constructs, no cross-loading exceeded .39, and every item with a lower primary loading belonged to the identity scale. This pattern, together with the higher-order confirmatory specification reported in Section 2, supports treating the five constructs as empirically distinguishable while representing identity through its three-dimensional higher-order structure.

Appendix Table 25

Varimax-rotated loadings of the forced five-factor principal axis solution.

Item	Factor 1	Factor 2	Factor 3	Factor 4	Factor 5
Multilingual social grooming					
Item 1	.68	.16	.23	.11	.17
Item 2	.68	.21	.24	.14	.18
Item 3	.70	.20	.27	.10	.22
Item 4	.72	.19	.30	.09	.13
Item 5	.69	.19	.27	.11	.15
Item 6	.66	.18	.31	.11	.22
Item 7	.65	.23	.26	.14	.15
Item 8	.62	.18	.24	.15	.19
Authentic pride					
Item 1	.17	.66	.15	.16	.19
Item 2	.20	.65	.13	.16	.14
Item 3	.25	.68	.14	.15	.19
Item 4	.20	.67	.17	.15	.18
Item 5	.18	.72	.20	.10	.25
Item 6	.20	.63	.16	.12	.20
Item 7	.21	.64	.18	.14	.16
Online bridging social capital					
Item 1	.29	.18	.69	.10	.20
Item 2	.27	.13	.70	.14	.18
Item 3	.19	.13	.71	.11	.17
Item 4	.23	.15	.64	.07	.23
Item 5	.23	.21	.69	.09	.21
Item 6	.25	.15	.68	.12	.20
Item 7	.23	.14	.68	.08	.21
Item 8	.23	.17	.67	.07	.17
Item 9	.27	.12	.72	.14	.15
Need for uniqueness					
Item 1	.06	.10	.08	.81	.07
Item 2	.13	.13	.11	.81	.09
Item 3	.10	.10	.09	.76	.07
Item 4	.14	.14	.13	.81	.14
Self-perceived multilingual identity					
Item 1	.30	.37	.27	.13	.32
Item 2	.31	.38	.32	.06	.35
Item 3	.33	.37	.30	.04	.37
Item 4	.39	.38	.35	.06	.34
Item 5	.37	.35	.34	.06	.32
Item 6	.31	.30	.33	.15	.32
Item 7	.25	.29	.28	.10	.65
Item 8	.19	.19	.27	.14	.65
Item 9	.20	.24	.25	.14	.67
Item 10	.25	.29	.23	.16	.60
Item 11	.20	.20	.30	.17	.62
Item 12	.20	.19	.25	.10	.62
Item 13	.35	.29	.38	.14	.23
Item 14	.31	.34	.39	.20	.22
Item 15	.36	.31	.37	.09	.23
Item 16	.33	.36	.39	.11	.23
Sum of squared loadings	5.94	5.20	6.66	3.15	4.20
Percentage of variance	13.5%	11.8%	15.1%	7.2%	9.6%

Note. Factor 1 = multilingual social grooming; Factor 2 = authentic pride; Factor 3 = online bridging social capital; Factor 4 = need for uniqueness; Factor 5 = self-perceived multilingual

identity. Cumulative variance explained = 57.2%. Item numbering follows Appendix Tables 2 through 6.

8. Reproducibility

8.1 Software, seeds, and supplied files

The primary analysis pipeline used Python 3.12.3 with NumPy 2.4.4, pandas 3.0.2, statsmodels 0.14.6, SciPy 1.17.1, and semopy 2.3.11. Primary indirect associations used 5,000 case-resampling percentile bootstrap samples with seed 20260718; supplementary primary-model analyses used seed 20260719. The independent relational-moderation audit used 10,000 case-resampling percentile bootstrap samples with seed 20260720 and reported HC3 covariance.

The supporting package contains the original analysis scripts and results file, the independent audit script and results, the revised figure-generation script, and all six 400-dpi PNG figures. The analytic data contain 755 retained cases and 50 columns, with no missing cells, exact duplicate rows, or duplicate 44-item response patterns. The analytic data do not contain the attention-check variable or the 30 excluded cases, so the exclusion flow cannot be independently reconstructed from that file. The screening counts are as reported by the survey company.

9. Independent-sample validation of the multilingual social grooming measure

9.1 Purpose and scope

The eight multilingual social grooming items were developed for this research and, in Study 1, were evaluated in the same sample that was used for the substantive analyses ($N = 755$). To obtain measurement evidence that does not depend on that sample, the eight items were administered to a second, independently recruited sample of Chinese university students in a separate measurement study. Item wording, item order, the three-month reference period and the seven-point frequency format were locked to the Chinese version administered in Study 1. The independent sample was collected for measurement purposes only. It did not include the authentic pride, online bridging social capital, need for uniqueness or self-perceived multilingual identity measures, and it was not used to estimate or re-estimate any association reported in the main text.

The validation was organised around nine questions (VQ1 to VQ9) specified before the analysis: sample independence and data quality (VQ1), item functioning (VQ2), the prespecified single-factor structure (VQ3), reliability and convergent validity (VQ4), comprehension and clarity of the items (VQ5), relations with single-item indicators of neighbouring behaviours and proficiency (VQ6), replication of the Study 1 psychometric profile (VQ7), measurement equivalence across gender (VQ8) and description of the sample (VQ9). Appendix Table 34 summarises the evidence for each question. In keeping with the confirmatory purpose of the study, no item was deleted, reworded or reordered on the basis of the results, and no exploratory factor analysis was used to modify the item set.

9.2 Participants and procedure

Participants were recruited and compensated by Market Research Company, which administered the online questionnaire. The research team supplied the eligibility criteria, the questionnaire, the informed-consent text and the data-quality requirements. Eligibility was identical to Study 1: participants had to be at least 18 years old, currently enrolled at a college or university in mainland China, regular users of at least one social media platform, and users of English or mixed Chinese-English communication at least once during the previous three months in a social media post, caption, comment, or public or semi-public reply. Independence from Study 1 was enforced by the firm through de-duplication of non-identifying panel identifiers, so that no participant in Study 1 could take part. The questionnaire contained one instructed-response attention check ("please select 4"). Following the Study 1 protocol, no completion-time threshold was applied.

The firm delivered 220 complete questionnaires. All 220 delivered cases satisfied the four eligibility screens and the attention check, had unique identifiers, had complete data on the eight grooming items and used only the permitted response categories. A careless-responding and outlier screen on the eight items, with rules fixed in advance (Meade & Craig, 2012; Tabachnick & Fidell, 2019), flagged no case: no respondent gave the same answer to all eight items, no two respondents shared an identical eight-item response pattern, no composite score lay beyond three standard deviations of the mean, and no respondent was a multivariate outlier (largest Mahalanobis distance 22.72 against a critical value of 26.12 at $p < .001$). The median longest run of identical consecutive answers was two. All 220 cases were therefore retained.

Appendix Table 26 describes the sample. Participants were 18 to 29 years old ($M = 20.32$, $SD = 2.21$), 48.6% men and 50.5% women, and 93.2% Han; 15 participants (6.8%) belonged to an ethnic minority (Tujia, Hui or Manchu). Three quarters were undergraduates and the remainder were junior-college, master's or doctoral students, at institutions in Chongqing, Sichuan, Shanghai, Beijing and Guangdong. Self-rated English proficiency ($M = 4.12$, $SD = 1.19$ on the seven-point scale) was close to the Study 1 mean of 4.15.

Appendix Table 26
Characteristics of the independent validation sample.

Characteristic	Category	*n*	%
Age (years)	*M* = 20.32, *SD* = 2.21, range 18-29		
Gender	Men	107	48.6
	Women	111	50.5
	Non-binary or other	2	0.9
Ethnicity	Han	205	93.2
	Hui	4	1.8
	Manchu	3	1.4
	Tujia	8	3.6
Degree level	Junior college	27	12.3

Characteristic	Category	*n*	%
	Undergraduate	164	74.5
	Master's	25	11.4
	Doctoral	4	1.8
Year of study	Year 1	66	30.0
	Year 2	63	28.6
	Year 3	59	26.8
	Year 4	32	14.5
Field of study	Humanities and social sciences	36	16.4
	Science, engineering, IT	51	23.2
	Medicine, agriculture, life sciences	38	17.3
	Arts or sports	55	25.0
	Other	40	18.2
Province of institution	Beijing	30	13.6
	Chongqing	70	31.8
	Guangdong	21	9.5
	Shanghai	35	15.9
	Sichuan	64	29.1
Institution type	Public undergraduate	132	60.0
	Private undergraduate	33	15.0
	Public junior college	18	8.2
	Private junior college	16	7.3
	Other	13	5.9
	Do not know	8	3.6
Home language(s) before age 18	Mandarin	171	77.7
	Chinese regional dialect	95	43.2
	Ethnic minority language	56	25.5
Language(s) with parents now	Mandarin	146	66.4
	Chinese regional dialect	146	66.4
Languages studied besides Chinese	None besides Chinese	58	26.4
	One	120	54.5
	Two	26	11.8
	Three or more	16	7.3
Daily social media time	< 30 min	35	15.9
	30-59 min	32	14.5
	1-2 h	45	20.5
	2-3 h	33	15.0
	3-4 h	42	19.1

Characteristic	Category	*n*	%
	> 4 h	33	15.0
Platforms used regularly	WeChat	220	100.0
	Weibo	109	49.5
	Xiaohongshu	128	58.2
	Douyin	128	58.2
	Bilibili	123	55.9
Most recent standardised English test	None or not provided	43	19.5
	CET-4	83	37.7
	CET-6	60	27.3
	IELTS	13	5.9
	TOEFL	6	2.7
	Other	15	6.8
Self-rated English proficiency (B5, 1-7)	*M* = 4.12, *SD* = 1.19		
Years of English study (B6)	*M* = 12.25, *SD* = 2.85, range 5-22		

Note. *N* = 220. Percentages for home languages, languages used with parents and platforms are based on multiple-response items and do not sum to 100. Ethnic minority participants: Tujia 8, Hui 4, Manchu 3. Self-rated proficiency and years of English study are reported as means and standard deviations.

9.3 Measures

Multilingual social grooming. The eight locked items (Appendix Table 2 gives the complete wording) were answered on the Study 1 frequency scale from 1 (never) to 7 (daily or almost daily) with reference to the previous three months. The composite score was the item mean.

Comprehension and clarity checks. Six closed items followed the grooming block and were not scored with it. Q1 asked whether the eight items were easy to understand, Q2 whether they were easy to distinguish from one another, and Q3 whether the frequency options could express the respondent's actual frequency, each on a five-point scale. Q4 asked respondents to mark any item that was difficult to understand (multiple response). Q5 was a knowledge check asking which of four behaviours the instructions counted as grooming, with one correct option (English or mixed Chinese-English in public or semi-public posts, comments or replies). Q6 asked which communication settings respondents had in mind when answering (posts or captions; comments or public or semi-public replies; fully private one-to-one chat; browsing only; multiple response).

Related fact indicators. Five single-item indicators were included to examine whether the grooming score is distinct from neighbouring behaviours and from proficiency: overall posting frequency in any language (B1) and overall commenting or replying frequency in any language (B2) on the same seven-point frequency scale; frequency of browsing English or mixed content without posting, commenting or replying (B3); frequency of English or mixed use in fully private one-to-one chat (B4); and self-rated English proficiency (B5, 1 = very limited to 7 = very

proficient). Years of English study (B6) and the score on the most recent standardised English test (B8, with test type recorded in B7) were recorded as exploratory indicators. These indicators were analysed individually and were not combined into a scale.

9.4 Analysis

Analyses were conducted in R 4.3.3 (R Core Team, 2024) with lavaan 0.6-17 (Rosseel, 2012) and psych 2.4.1 (Revelle, 2024); the script, its console log and all result tables accompany the manuscript. Before analysis the eight items were screened for careless responding and multivariate outliers with rules fixed in advance: the instructed-response attention check, duplicate identifiers and duplicate eight-item response patterns, straightlining, composite scores beyond three standard deviations, and Mahalanobis distance at $p < .001$ (Meade & Craig, 2012; Tabachnick & Fidell, 2019). The prespecified single-factor model for the eight items was estimated with robust maximum likelihood (MLR), and fit was evaluated with the Yuan-Bentler scaled chi-square, the robust comparative fit index, Tucker-Lewis index and root-mean-square error of approximation with its 90% confidence interval, and the standardised root-mean-square residual, against the conventional criteria of Hu and Bentler (1999). Ninety-five per cent confidence intervals for standardised loadings were obtained by the delta method and, as a check, by percentile bootstrap with 2,000 case resamples; the bootstrap was also used for composite reliability and average variance extracted. Two supplementary models were fitted: a weighted least squares (WLSMV) model treating the items as ordinal, and a correlated three-facet model (self-disclosure, MSG1 and MSG2; responsive interaction, MSG3 to MSG6; relationship maintenance, MSG7 and MSG8) used to check whether the content facets are empirically separable, with the scaled chi-square difference test of Satorra and Bentler (2001). Parallel analysis (Horn, 1965; 500 iterations), the Kaiser-Meyer-Olkin statistic and Bartlett's test served as descriptive dimensionality checks. Internal consistency was summarised by Cronbach's alpha with Feldt (1965) and bootstrap confidence intervals, McDonald's omega from the confirmatory solution, composite reliability and average variance extracted (Fornell & Larcker, 1981).

Comprehension checks were summarised descriptively, and the confirmatory model and reliability were re-estimated in the subsample that answered the knowledge check correctly and did not report thinking of private chat or browsing only. Associations between the grooming score and the fact indicators were examined as zero-order correlations with Fisher confidence intervals, as correlations between the latent grooming factor and the single-indicator variables, and as partial correlations. The psychometric profile was compared descriptively with the Study 1 values in Appendix Tables 2 and 9. Measurement equivalence across gender was tested with configural, metric and scalar multigroup models, using changes in the comparative fit index of .010 or less and in the root-mean-square error of approximation of .015 or less as evidence of invariance (Chen, 2007; Cheung & Rensvold, 2002). Ethnicity, degree level and province were described but not tested because their subgroups were too small for multigroup analysis.

9.5 Results

9.5.1 Item functioning (VQ2)

Appendix Table 27 reports the item statistics. Every item used the full 1 to 7 range, item means ranged from 3.64 (MSG7) to 4.66 (MSG3), standard deviations from 1.34 to 1.48, skewness from −.18 to .04 and kurtosis from −.66 to −.27. No floor category exceeded 6.8% and no ceiling category exceeded 10.5%. Corrected item-total correlations ranged from .53 (MSG8) to .71 (MSG4), inter-item correlations from .32 to .59 (mean .44), and deleting any single item lowered Cronbach's alpha (alpha if deleted .836 to .856 against .864 for the full set). The composite score had a mean of 4.21 (*SD* = 1.00; range 1.62 to 6.50; skewness −.16; kurtosis −.52).

Appendix Table 27
Item-level statistics for the eight multilingual social grooming items in the independent sample.

Item	*M*	*SD*	Skewness	Kurtosis	Floor %	Ceiling %	Corrected item-total *r*	Alpha if item deleted
MSG1	4.55	1.36	−.14	−.45	0.9	7.7	.61	.848
MSG2	4.20	1.48	−.05	−.66	2.7	5.9	.65	.844
MSG3	4.66	1.34	−.14	−.27	0.9	10.5	.56	.854
MSG4	4.03	1.45	−.05	−.58	3.6	4.1	.71	.836
MSG5	3.81	1.44	.04	−.47	5.0	3.6	.67	.841
MSG6	4.48	1.41	−.18	−.46	1.8	7.3	.61	.848
MSG7	3.64	1.35	−.12	−.53	6.8	0.5	.56	.854
MSG8	4.33	1.38	−.04	−.31	2.3	6.4	.53	.856

Note. N = 220. Item wording follows Appendix Table 2 (MSG1 share daily experiences; MSG2 express thoughts and feelings; MSG3 acknowledge, encourage or support others; MSG4 comment on others' posts; MSG5 reply to comments on own posts; MSG6 greetings, wishes and brief supportive messages; MSG7 keep in touch with people not often seen in person; MSG8 maintain relationships). Floor and ceiling give the percentage of respondents choosing 1 (never) and 7 (daily or almost daily). Skewness and kurtosis use the sample-adjusted formulas reported for Study 1. Cronbach's alpha for all eight items = .864.

9.5.2 Factor structure (VQ3)

Sampling adequacy was high (Kaiser-Meyer-Olkin = .909, lowest item value .89; Bartlett's chi-square(28) = 623.15, $p < .001$). The eigenvalues of the item correlation matrix were 4.11, .75, .69, .61, .57, .46, .43, .38, giving a first-to-second ratio of 5.48, and parallel analysis retained one factor and one component. The prespecified single-factor model fit the data well: scaled chi-square(20) = 24.46, $p = .223$; comparative fit index = .993; Tucker-Lewis index = .990; root-mean-square error of approximation = .032, 90% CI [.000, .070]; standardised root-mean-square residual = .031 (Appendix Table 29). Standardised loadings ranged from .57 (MSG8) to .78 (MSG4), and every 95% confidence interval excluded .40 by both the delta method and the percentile bootstrap (lowest lower bound .47; Appendix Table 28 and Appendix Figure 2). The largest absolute standardised residual correlation was .07 (MSG5 with MSG7) and the largest modification index was 4.83 (residual covariance between MSG5 and MSG7), so no local misfit was indicated. Treating the items as ordinal (WLSMV) gave the same conclusion (comparative fit

index = .990; root-mean-square error of approximation = .039; loadings .59 to .80).

The correlated three-facet model also fit well, but its facet correlations were 1.00 (self-disclosure with responsive interaction), .98 (responsive interaction with relationship maintenance) and .84 (self-disclosure with relationship maintenance), and the scaled chi-square difference against the single-factor model was not significant (difference = 6.37, *df* = 3, *p* = .095). The content facets are therefore not empirically separable, which supports scoring the eight items as a single composite, as in Study 1.

Appendix Table 28

Standardised loadings of the single-factor model in the independent sample, with Study 1 values for comparison.

Item	Loading (MLR)	95% CI, delta method	95% CI, bootstrap	R^2	WLSMV loading	Study 1 loading	Study 1 corrected item-total *r*
MSG1	.68	[.59, .76]	[.59, .75]	.46	.69	.76	.73
MSG2	.71	[.65, .79]	[.64, .78]	.51	.72	.79	.75
MSG3	.60	[.51, .70]	[.50, .69]	.36	.63	.82	.78
MSG4	.78	[.71, .85]	[.70, .84]	.60	.80	.81	.78
MSG5	.73	[.65, .81]	[.65, .80]	.53	.75	.79	.76
MSG6	.65	[.57, .74]	[.56, .74]	.43	.67	.80	.76
MSG7	.60	[.50, .69]	[.50, .69]	.36	.62	.77	.74
MSG8	.57	[.47, .66]	[.47, .66]	.32	.59	.73	.70

Note. Independent sample *N* = 220; Study 1 *N* = 755. Study 1 values are those reported in Appendix Table 2; the Study 1 loadings were estimated within the five-construct confirmatory model, whereas the independent-sample loadings come from a stand-alone single-factor model. Bootstrap intervals are percentile intervals from 2,000 case resamples of the maximum likelihood solution.

Appendix Table 29

Fit of the confirmatory models for the eight grooming items in the independent sample.

Model	Scaled χ^2	*df*	*p*	CFI	TLI	RMSEA [90% CI]	SRMR	*N*
One factor, robust ML (primary)	24.46	20	.223	.993	.990	.032 [.000, .070]	.031	220
One factor, WLSMV, items ordinal (sensitivity)	26.66	20	.145	.990	.986	.039 [.000, .080]	.034	220
Three correlated facets, robust ML (scoring check)	18.09	17	.383	.998	.997	.017 [.000, .065]	.026	220
One factor, robust ML, comprehension-consistent subsample	28.50	20	.098	.984	.977	.050 [.000, .089]	.036	169

Note. CFI = comparative fit index; TLI = Tucker-Lewis index; RMSEA = root-mean-square error of approximation; SRMR = standardised root-mean-square residual. Robust (MLR) or scaled (WLSMV) versions are reported. Facet correlations in the three-facet model: self-disclosure with responsive interaction 1.00; self-disclosure with relationship maintenance .84; responsive interaction with relationship maintenance .98. The comprehension-consistent subsample excludes respondents who answered the Q5 knowledge check incorrectly or reported thinking of private chat or browsing only in Q6.

9.5.3 Reliability and convergent validity (VQ4)

Cronbach's alpha was .864, 95% CI [.835, .890] (Feldt) and [.835, .886] (bootstrap); McDonald's omega from the confirmatory solution was .866 and composite reliability was .865, 95% CI [.839, .887]. Average variance extracted was .446, 95% CI [.398, .499], and .471 in the WLSMV solution (composite reliability .876). With composite reliability well above .60 and all loadings significant and above .50, convergent validity is adequate under the criterion of Fornell and Larcker (1981). Appendix Table 30 places these values beside the Study 1 estimates.

Appendix Table 30

Reliability and convergent validity of the grooming measure in the independent sample and in Study 1.

Statistic	Independent sample (*N* = 220)	Study 1 (*N* = 755)
Cronbach's alpha [95% CI, Feldt]	.864 [.835, .890]	.927
McDonald's omega (confirmatory solution)	.866	—
Composite reliability [95% bootstrap CI]	.865 [.839, .887]	.927
Average variance extracted [95% bootstrap CI]	.446 [.398, .499]	.615
Composite reliability, WLSMV solution	.876	—
Average variance extracted, WLSMV solution	.471	—
Mean inter-item correlation	.442	—
Range of standardised loadings	.57–.78	.73–.82
Range of corrected item-total correlations	.53–.71	.70–.78
Composite score *M* (*SD*)	4.21 (1.00)	4.64 (1.32)

Note. Study 1 values are those reported in Appendix Tables 2, 7 and 9. Dashes indicate statistics that were not reported for Study 1.

9.5.4 Comprehension and clarity (VQ5)

Appendix Table 31 summarises the six checks. Respondents found the items easy to understand (Q1: *M* = 4.30, 87.7% rating 4 or 5, none rating 1 or 2), easy to distinguish from one another (Q2: *M* = 4.08, 76.8% rating 4 or 5, 1.8% rating 1 or 2) and judged the frequency options able to express their actual frequency (Q3: *M* = 4.35, 94.5% rating 4 or 5). In Q4, 85.9% reported no difficult item, and no item was marked by more than 5.5% of respondents (MSG3, MSG5, MSG6 and MSG7 were not marked by anyone). In the Q5 knowledge check, 85.0% selected the correct scope (English or mixed use in public or semi-public posts, comments or replies). In Q6, respondents mainly had public production in mind (posts or captions 68.6%, comments or public replies 60.0%); 15 respondents (6.8%) also named fully private chat and 10 (4.5%) browsing only.

Re-estimating the model in the 169 respondents who answered Q5 correctly and did not name private chat or browsing only in Q6 left the conclusions unchanged: scaled chi-square(20) = 28.50, comparative fit index = .984, Tucker-Lewis index = .977, root-mean-square error of approximation = .050, standardised root-mean-square residual = .036, loadings .60–.79, alpha = .875, composite reliability = .875 and average variance extracted = .469 (Appendix Table 29). The measurement

results therefore do not depend on respondents' interpretation of the instructions.

Appendix Table 31

Comprehension and clarity checks for the grooming items in the independent sample.

Check	Item or category	*n*	%	*M* (*SD*)
Clarity ratings (1 to 5)	Q1. Items easy to understand (rated 4 or 5)	193	87.7	4.30 (0.68)
	Q2. Items easy to distinguish (rated 4 or 5)	169	76.8	4.08 (0.80)
	Q3. Response options fit actual frequency (rated 4 or 5)	208	94.5	4.35 (0.58)
Q4. Items marked as difficult to understand	No item marked	189	85.9	
	MSG1	12	5.5	
	MSG2	9	4.1	
	MSG3	0	0.0	
	MSG4	9	4.1	
	MSG5	0	0.0	
	MSG6	0	0.0	
	MSG7	0	0.0	
	MSG8	10	4.5	
Q5. Knowledge check: which behaviour counts	Browsing English posts only	12	5.5	
	English in fully private chat	12	5.5	
	English or mixed in public/semi-public posts, comments, replies (correct)	187	85.0	
	English course assignments	9	4.1	
Q6. Settings respondents had in mind	Posts or captions	151	68.6	
	Comments or public/semi-public replies	132	60.0	
	Fully private one-to-one chat	15	6.8	
	Browsing others' content only	10	4.5	

Note. $N = 220$. Q1 to Q3 were rated from 1 (very difficult to understand; cannot distinguish at all; cannot express at all) to 5 (very easy to understand; very easy to distinguish; can express completely); *n* and % give the respondents rating 4 or 5. Q4 and Q6 allowed multiple responses. The correct Q5 option is the third.

9.5.5 Relations with fact indicators (VQ6)

Appendix Table 32 reports the associations. The grooming score correlated positively but moderately with overall posting frequency ($r = .33$), overall commenting frequency ($r = .42$), browsing English content ($r = .26$), English use in private chat ($r = .31$) and self-rated proficiency ($r = .49$); the corresponding latent correlations ranged from .28 to .52, all well below .85. Self-rated proficiency retained a partial correlation of .41 with grooming after posting and commenting frequency were controlled, and posting (.24) and commenting (.33) retained positive partial

correlations after proficiency was controlled, so the measure is neither a proxy for general social media activity nor a proxy for proficiency. Browsing English content had no unique association with grooming once activity and proficiency were controlled (partial $r = -.00$, $p = .957$), and private chat had only a small, non-significant unique association (partial $r = .13$, $p = .052$). This pattern matches the construct definition, which excludes passive consumption and private messaging. Among the exploratory indicators, grooming correlated .33 with years of English study and .46 ($n = 156$) with the most recent standardised test score expressed as a z score within test type (CET-4, CET-6 and IELTS), a proficiency indicator that does not rely on self-rating.

Appendix Table 32

Associations between the grooming score and the single-item fact indicators in the independent sample.

Indicator	Description	*n*	Zero-order *r*	95% CI	Latent *r* [95% CI]	Partial *r*	Controlling for
B1	Overall posting frequency (any language)	220	.33	[.20, .44]	.36 [.24, .48]	.24	B5
B2	Overall commenting/replying frequency (any language)	220	.42	[.30, .52]	.46 [.35, .57]	.33	B5
B3	Browsing English or mixed content only	220	.26	[.14, .38]	.28 [.15, .42]	−.00 (ns)	B1, B2, B5
B4	English or mixed use in fully private chat	220	.31	[.19, .43]	.34 [.21, .47]	.13 (ns)	B1, B2, B5
B5	Self-rated English proficiency	220	.49	[.38, .58]	.52 [.41, .63]	.41	B1, B2
B6	Years of English study	220	.33	[.20, .44]	—	—	—
B8z	Most recent test score, z within test type (exploratory)	156	.46	[.32, .57]	—	—	—

Note. Latent correlations come from a model in which the eight grooming items load on one factor and B1 to B5 enter as single-indicator variables (scaled chi-square(55) = 68.11, comparative fit index = .985, root-mean-square error of approximation = .033). Partial correlations are significant at $p < .001$ unless marked ns (B3: $p = .957$; B4: $p = .052$). B8 was standardised within test type for test types with at least ten scores (CET-4, CET-6, IELTS). B1 to B5 are separate indicators and were not combined into a scale.

9.5.6 Comparison with Study 1 (VQ7)

The independent sample led to the same conclusions as Study 1: a single factor with good fit, all loadings above .50, all corrected item-total correlations above .40, and alpha and composite reliability above .85 (Appendix Tables 28 and 30). Loadings and reliability were somewhat lower than in Study 1 (loadings .57 to .78 against .73 to .82; alpha .86 against .93). Grooming scores in the independent sample were lower and less dispersed than in Study 1 (composite $M = 4.21$, $SD = 1.00$, against 4.64, $SD = 1.32$; item standard deviations 1.34 to 1.48 against 1.52 to 1.68), and a narrower distribution attenuates inter-item correlations; in addition, the Study 1 loadings were estimated within the five-construct model rather than as a stand-alone factor. The independent-sample values can be regarded as a conservative estimate of the measure's item quality.

9.5.7 Measurement equivalence across gender (VQ8)

Appendix Table 33 reports the multigroup models for men ($n = 107$) and women ($n = 111$). The

configural model fit well, constraining the loadings to equality did not worsen fit (scaled chi-square difference = 9.51, $df = 7$, $p = .218$; change in comparative fit index = −.004; change in root-mean-square error of approximation = .004), and adding equal intercepts likewise did not (scaled chi-square difference = 4.63, $df = 7$, $p = .705$; change in comparative fit index = .004; change in root-mean-square error of approximation = −.009). Full scalar invariance across gender was therefore supported, so the measure can be compared across men and women. Under the scalar model the latent mean did not differ significantly between men and women (difference = .22, $SE = .13$, $p = .091$). Descriptively, composite means were similar across ethnicity (Han 4.23, ethnic minority 3.97, $n = 15$), provinces (4.10 to 4.42) and degree levels (4.12 to 4.66); these subgroups were too small for invariance testing.

Appendix Table 33

Measurement invariance of the single-factor grooming model across gender in the independent sample.

Model	Scaled χ^2	*df*	*p*	CFI	RMSEA [90% CI]	SRMR	Scaled $\Delta\chi^2$ (Δdf)	*p* (Δ)	ΔCFI	ΔRMSEA
Configural	44.18	40	.299	.993	.031 [.000, .075]	.039	—	—	—	—
Metric (equal loadings)	53.54	47	.238	.989	.035 [.000, .074]	.059	9.51 (7)	.218	−.004	.004
Scalar (equal loadings and intercepts)	58.18	54	.324	.993	.026 [.000, .067]	.061	4.63 (7)	.705	.004	−.009

Note. Men $n = 107$, women $n = 111$; Tucker-Lewis index = .990, .987 and .993 for the three models; two respondents who identified as non-binary or other were not included. Differences are relative to the preceding model. Invariance criteria: ΔCFI ≤ .010 and ΔRMSEA ≤ .015 (Chen, 2007; Cheung & Rensvold, 2002).

9.5.8 Summary

Appendix Table 34 summarises the evidence for the nine validation questions. In a sample that played no part in hypothesis testing, the eight items formed a single factor with good fit and no local misfit, all loadings and corrected item-total correlations exceeded conventional thresholds with confidence intervals that excluded .40, internal consistency was high, the composite scoring used in Study 1 was supported over a facet structure, the items were understood as intended by the large majority of respondents, the measure was invariant across gender, and the grooming score was related to, but distinct from, general posting and commenting activity, passive consumption of English content, private English messaging and English proficiency. The independent sample therefore reproduces the structure, reliability and scoring of the multilingual social grooming measure.

Appendix Table 34

Summary of the validation evidence by validation question.

VQ	Evidence	Result
VQ1	Cases retained after eligibility, attention-check, duplicate, completeness and outlier screens	220 of 220 (no case flagged by any screen)
VQ2	Item skewness and kurtosis (largest absolute values)	|skewness| ≤ .18, |kurtosis| ≤ .66

VQ	Evidence	Result
	Corrected item-total correlations	.53 to .71
	Inter-item correlations	.32 to .59 (mean .44)
VQ3	Single-factor model fit (MLR)	CFI = .993, TLI = .990, RMSEA = .032 [.000, .070], SRMR = .031
	Standardised loadings (lowest 95% CI bound)	.57 to .78 (lowest bound .47)
	Three-facet check: facet correlations; scaled chi-square difference vs one factor	r = .84 to 1.00; diff = 6.37, df = 3, p = .095
VQ4	Cronbach's alpha [95% CI]	.864 [.835, .890]
	McDonald's omega; composite reliability	.866; .865
	Average variance extracted [95% bootstrap CI]	.446 [.398, .499]
VQ5	Clarity ratings Q1-Q3 (mean; % rating 4 or 5)	4.3 / 4.08 / 4.35; 87.7 / 76.8 / 94.5%
	Q5 knowledge check correct	85.0%
	Comprehension-consistent subsample: fit, alpha, loadings	n = 169: CFI = .984, alpha = .875, loadings .60 to .79
VQ6	Correlations with B1-B5 (zero-order; latent)	r = .26 to .49; latent r = .28 to .52
	Partial correlations: B5 | B1, B2; B1 | B5; B2 | B5	.41; .24; .33
VQ7	Same acceptability decisions as Study 1 (loadings ≥ .50, CITC ≥ .40, alpha ≥ .80)	loadings 8 of 8; CITC 8 of 8; alpha .86
VQ8	Gender invariance: change in CFI and RMSEA at metric and scalar steps	ΔCFI = −.004, .004; ΔRMSEA = .004, −.009

Note. VQ1 sample independence and data quality; VQ2 item functioning; VQ3 single-factor structure; VQ4 reliability and convergent validity; VQ5 comprehension and clarity; VQ6 relations with fact indicators; VQ7 replication of the Study 1 profile; VQ8 gender equivalence; VQ9 sample description (Appendix Table 26). Correlations, fit indices, loadings and reliabilities are shown without leading zeros.

Appendix Figure 2

Standardised loadings of the eight grooming items in the independent sample (with 95%

confidence intervals) and in Study 1.

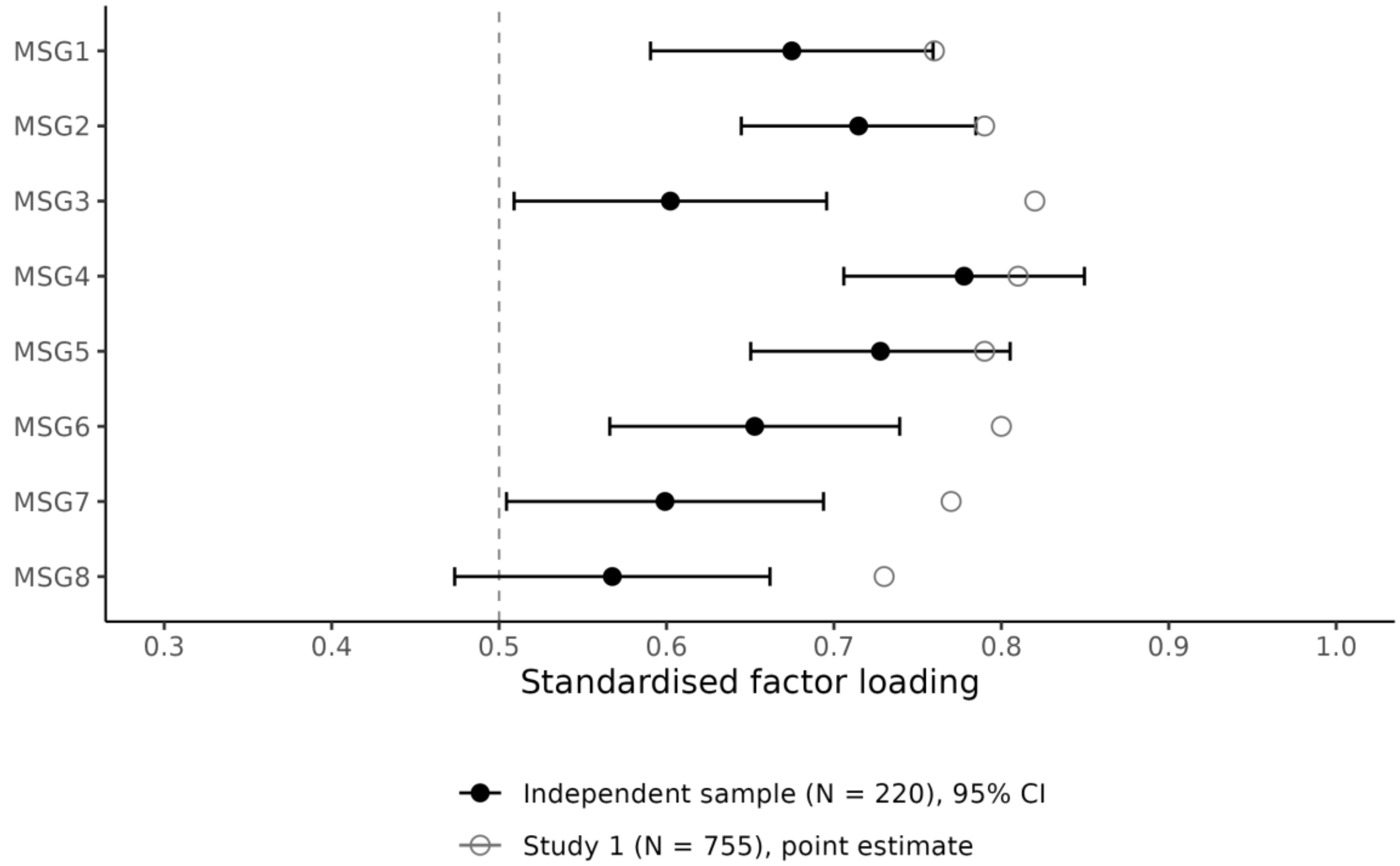

Note. Filled circles with error bars are robust maximum likelihood estimates and delta-method 95% confidence intervals in the independent sample ($N = 220$); open circles are the Study 1 point estimates from Appendix Table 2 ($N = 755$). The dashed line marks the .50 loading criterion.

9.6 Scope of the evidence

The independent sample provides external measurement evidence for the grooming instrument: its structure, reliability and composite scoring were reproduced in a sample that played no part in hypothesis testing, and the measure was shown to be distinct from neighbouring behaviours and from proficiency. As a measurement study it does not bear on the single-source design of the Study 1 structural model, and the cross-sectional associations of Study 1 remain associational. Test-retest reliability and criterion-related validity against behavioural records of multilingual posting are natural next steps for the instrument.

10. City tier, ethnicity, and English proficiency: supplementary analyses

10.1 Purpose and data

The survey company supplied two participant characteristics that were not analysed in the original submission: the city tier of each participant's institution (first- to fourth-tier city) and ethnicity (Han or ethnic minority). This section reports their distribution, compares the composite scores across these groups, re-estimates the primary conditional process model with the two variables added as controls, examines English proficiency as a moderator of the grooming associations, and reports Cohen's f^2 for every interaction term. All analyses use the 755 retained cases and were

conducted in R 4.3.3 with HC3 inference and 5,000-sample percentile bootstrap intervals for indirect associations (seed 20260718); the primary model was first re-estimated in R and reproduced every estimate in Table 3, Table 4 and Appendix Table 15.

10.2 Distribution and group comparisons

Institutions were located in first-tier (172, 22.8%), second-tier (223, 29.5%), third-tier (239, 31.7%) and fourth-tier (121, 16.0%) cities; 40 participants (5.3%) were ethnic minority students, distributed across all tiers (5, 15, 16, 4 in tiers 1 to 4; chi-square(3) = 4.75, p = .191). Appendix Table 35 reports the composite means by city tier with Welch analyses of variance; none of the five focal composites or self-rated proficiency differed across tiers (all $p \geq .063$, eta-squared $\leq .009$), and Appendix Figure 3 displays the means. Appendix Table 36 compares Han and ethnic minority participants. Minority participants did not differ on grooming, pride, identity or need for uniqueness (|Hedges' g| ≤ .20), reported lower bridging capital (g = −.25, 95% CI [−.57, .07]) and higher self-rated English proficiency (g = .36, 95% CI [.04, .68]). With 40 minority and 715 Han participants, the smallest standardised difference detectable with 80% power was d = .46 (.53 with 90% power), so the comparisons rule out large differences only.

Appendix Table 35

Composite means by city tier of the institution.

Variable	Tier 1 (*n* = 172)	Tier 2 (*n* = 223)	Tier 3 (*n* = 239)	Tier 4 (*n* = 121)	Welch *F*	*df*	*p*	η²
Multilingual social grooming	4.53 (1.53)	4.64 (1.35)	4.73 (1.19)	4.62 (1.19)	0.68	3, 368.0	.565	.003
Authentic pride	4.45 (1.36)	4.68 (1.35)	4.67 (1.15)	4.40 (1.13)	2.45	3, 370.8	.063	.009
Online bridging social capital	4.47 (1.45)	4.58 (1.31)	4.72 (1.19)	4.55 (1.18)	1.26	3, 368.8	.288	.005
Need for uniqueness	4.44 (1.39)	4.46 (1.43)	4.36 (1.37)	4.43 (1.28)	0.19	3, 373.2	.900	.001
Self-perceived multilingual identity	4.43 (1.29)	4.56 (1.12)	4.59 (1.05)	4.39 (1.01)	1.30	3, 369.3	.273	.005
English proficiency	4.20 (1.28)	4.10 (1.21)	4.19 (1.28)	4.08 (1.33)	0.40	3, 365.1	.750	.002

Note. Cells are means with standard deviations in parentheses on the seven-point metric. Welch analysis of variance with Welch degrees of freedom; η² from the ordinary one-way analysis of variance.

Appendix Table 36

Composite means by ethnicity, and exploratory grooming-by-ethnicity interactions.

Variable	Han (*n* = 715)	Ethnic minority (*n* = 40)	Difference	Welch *t*	*df*	*p*	Hedges' *g*	95% CI for *g*
Multilingual social grooming	4.64 (1.32)	4.63 (1.36)	−0.01	−0.04	43.2	.969	−.01	[−.32, .31]
Authentic pride	4.58 (1.27)	4.56 (1.16)	−0.02	−0.11	44.4	.910	−.02	[−.34, .30]
Online bridging social capital	4.61 (1.28)	4.29 (1.39)	−0.32	−1.43	42.8	.161	−.25	[−.57, .07]
Need for uniqueness	4.43 (1.38)	4.16 (1.35)	−0.27	−1.23	43.7	.224	−.20	[−.52, .12]
Self-perceived multilingual identity	4.52 (1.12)	4.42 (1.12)	−0.09	−0.51	43.6	.611	−.08	[−.40, .24]
English proficiency	4.12 (1.27)	4.58 (1.11)	0.45	2.48	44.9	.017	.36	[.04, .68]

Note. Difference = minority minus Han. Minimum detectable standardised difference with 80% power: d = .46.

Exploratory grooming-by-ethnicity interactions, each added to the corresponding equation with city tier and ethnicity as controls (HC3 inference): pride *b* = .018, 95% CI [−.288, .325], *p* = .907; bridging capital *b* = .127, [−.139, .394], *p* = .349; identity *b* = .050, [−.062, .161], *p* = .381.

Appendix Figure 3

Composite means with 95% confidence intervals by city tier of the institution.

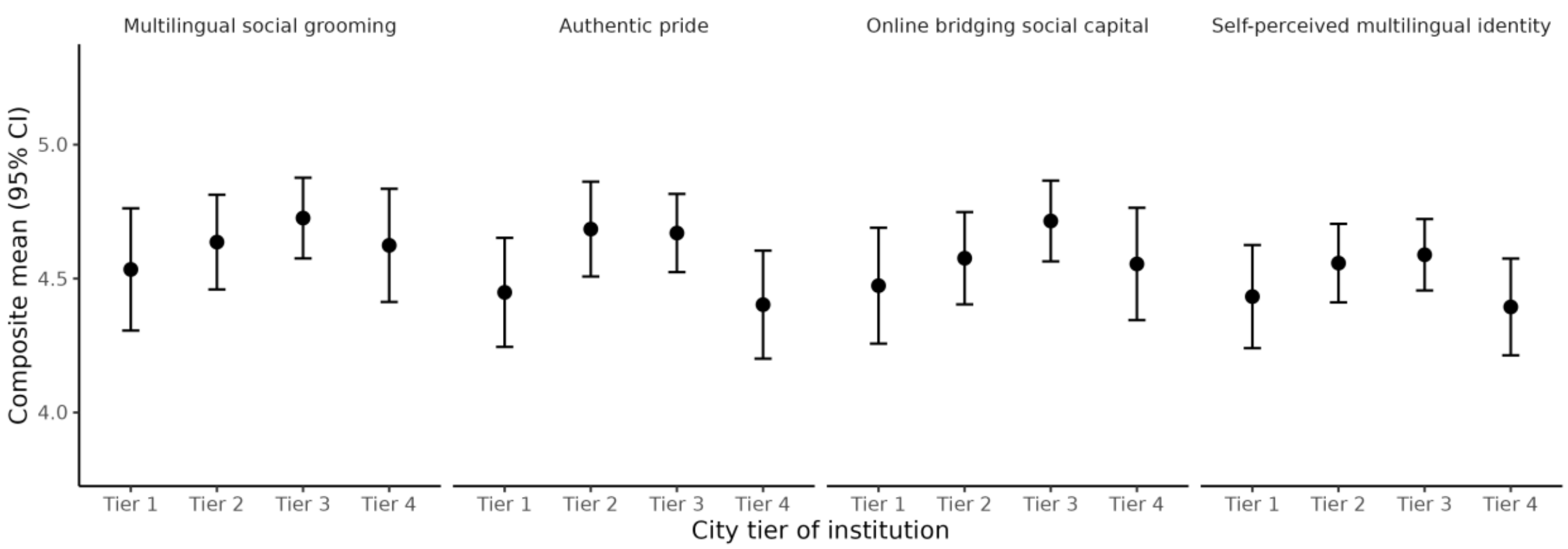

Note. N = 755. Confidence intervals are based on the standard error of the mean within each tier.

10.3 City tier and ethnicity as additional controls

Appendix Table 37 reports the seven focal coefficients of the primary model under five control specifications, and Appendix Table 38 the coefficients of the added controls. Every focal estimate was unchanged to the second decimal when city tier, ethnicity, or both were added, and when age, gender and field of study were added as well; R-squared rose by at most .011. City tier was jointly unrelated to bridging capital and identity and marginally related to pride (Appendix Table 38), and ethnicity was unrelated to all three outcomes. Appendix Table 39 shows that the indirect associations, the index of moderated indirect association and the exploratory relational interaction were likewise unchanged.

Appendix Table 37

Focal coefficients of the primary model under five control specifications.

Specification	Path	*b*	HC3 *SE*	95% HC3 CI	*p*
Primary controls	Pride: grooming	.461	.031	[.400, .522]	< .001
	Pride: need for uniqueness	.188	.027	[.134, .242]	< .001
	Pride: grooming x uniqueness	.044	.020	[.004, .084]	.031
	Bridging: grooming	.620	.028	[.565, .676]	< .001
	Identity: grooming (direct)	.224	.022	[.180, .268]	< .001
	Identity: pride	.277	.021	[.236, .317]	< .001
	Identity: bridging	.321	.022	[.279, .364]	< .001
Primary + city tier	Pride: grooming	.458	.031	[.397, .519]	< .001
	Pride: need for uniqueness	.190	.027	[.136, .243]	< .001
	Pride: grooming x uniqueness	.042	.021	[.002, .083]	.042

Specification	Path	*b*	HC3 *SE*	95% HC3 CI	*p*
	Bridging: grooming	.618	.028	[.563, .674]	< .001
	Identity: grooming (direct)	.225	.023	[.181, .269]	< .001
	Identity: pride	.275	.021	[.234, .316]	< .001
	Identity: bridging	.322	.022	[.279, .365]	< .001
Primary + ethnicity	Pride: grooming	.461	.031	[.400, .522]	< .001
	Pride: need for uniqueness	.188	.028	[.134, .242]	< .001
	Pride: grooming x uniqueness	.044	.021	[.004, .085]	.031
	Bridging: grooming	.619	.028	[.564, .675]	< .001
	Identity: grooming (direct)	.225	.023	[.180, .269]	< .001
	Identity: pride	.277	.021	[.236, .317]	< .001
	Identity: bridging	.320	.022	[.277, .363]	< .001
Primary + city tier + ethnicity	Pride: grooming	.458	.031	[.397, .519]	< .001
	Pride: need for uniqueness	.189	.027	[.135, .243]	< .001
	Pride: grooming x uniqueness	.042	.021	[.002, .083]	.042
	Bridging: grooming	.617	.028	[.562, .673]	< .001
	Identity: grooming (direct)	.225	.023	[.181, .270]	< .001
	Identity: pride	.275	.021	[.234, .316]	< .001
	Identity: bridging	.321	.022	[.278, .364]	< .001
Full extended (tier, ethnicity, age, gender, major)	Pride: grooming	.458	.031	[.396, .519]	< .001
	Pride: need for uniqueness	.189	.028	[.135, .243]	< .001
	Pride: grooming x uniqueness	.045	.021	[.004, .085]	.031
	Bridging: grooming	.616	.029	[.560, .672]	< .001
	Identity: grooming (direct)	.225	.023	[.181, .270]	< .001
	Identity: pride	.276	.021	[.235, .317]	< .001
	Identity: bridging	.320	.022	[.277, .364]	< .001

Note. All specifications include English proficiency, number of foreign languages studied and daily social media time. R-squared for pride, bridging capital and identity: Primary controls .369/.432/.762; Primary + city tier .375/.433/.763; Primary + ethnicity .369/.435/.763; Primary + city tier + ethnicity .376/.436/.763; full extended .380/.437/.763.

Appendix Table 38

City tier and ethnicity coefficients in the model with both added, and joint tests for city tier.

Criterion	Predictor	*b*	HC3 *SE*	95% HC3 CI	*p*
Authentic pride	City tier 2 vs tier 1	.192	.101	[−.007, .390]	.058
	City tier 3 vs tier 1	.162	.099	[−.032, .357]	.102
	City tier 4 vs tier 1	−.061	.116	[−.288, .166]	.598
	Ethnic minority vs Han	−.079	.150	[−.374, .217]	.602
Online bridging social capital	City tier 2 vs tier 1	.049	.106	[−.158, .257]	.640

Criterion	Predictor	*b*	HC3 *SE*	95% HC3 CI	*p*
	City tier 3 vs tier 1	.132	.100	[−.064, .329]	.185
	City tier 4 vs tier 1	.041	.112	[−.178, .260]	.711
	Ethnic minority vs Han	−.315	.168	[−.646, .015]	.061
Multilingual identity	City tier 2 vs tier 1	.026	.053	[−.078, .129]	.627
	City tier 3 vs tier 1	−.022	.056	[−.132, .088]	.691
	City tier 4 vs tier 1	−.047	.066	[−.177, .083]	.480
	Ethnic minority vs Han	−.070	.081	[−.230, .089]	.387

Note. Reference categories: first-tier city; Han. Joint HC3 Wald tests for the three city-tier coefficients: pride $F(3, 744) = 2.49$, $p = .059$; bridging capital $F(3, 746) = 0.68$, $p = .563$; identity $F(3, 744) = 0.55$, $p = .647$.

Appendix Table 39

Indirect associations and the exploratory relational interaction with and without city tier and ethnicity as controls.

Specification	Quantity	Estimate	95% CI
Primary controls	Indirect through pride	.128	[.104, .152]
	Indirect through bridging capital	.199	[.168, .232]
	Index of moderated indirect (pride)	.012	[.002, .024]
	Contrast: bridging minus pride	.072	[.033, .111]
	Conditional pride indirect, low uniqueness	.111	[.080, .140]
	Conditional pride indirect, high uniqueness	.144	[.117, .173]
Primary + city tier + ethnicity	Indirect through pride	.126	[.102, .151]
	Indirect through bridging capital	.198	[.167, .231]
	Index of moderated indirect (pride)	.012	[.002, .024]
	Contrast: bridging minus pride	.072	[.034, .112]
	Conditional pride indirect, low uniqueness	.110	[.078, .139]
	Conditional pride indirect, high uniqueness	.142	[.114, .171]
Primary controls	Grooming-by-uniqueness interaction in the bridging equation (HC3)	−.037	[−.065, −.008]
Primary + city tier + ethnicity	Grooming-by-uniqueness interaction in the bridging equation (HC3)	−.036	[−.064, −.007]

Note. Indirect quantities use 5,000-sample percentile bootstrap intervals; the relational interaction uses HC3 intervals. Low and high uniqueness are one standard deviation below and above the mean.

10.4 English proficiency as a moderator

Appendix Table 40 reports the grooming-by-proficiency interaction added to each equation (with need for uniqueness and its interaction retained in the pride equation, and number of languages and social media time as controls). Proficiency strengthened the grooming–pride association ($b = .070$, $p = .003$, $f^2 = .014$) and the direct grooming–identity association ($b = .033$, $p = .013$, $f^2 = .010$); the grooming–bridging association did not vary with proficiency ($p = .125$). Simple slopes were positive at every level of proficiency. Proficiency correlated .393 with identity, .225 with

pride and .141 with bridging capital, and retained unique associations with identity (b = .200) and pride (b = .127) in the primary equations; the grooming–identity correlation was .718 at zero order and .716 with proficiency partialled out.

Appendix Table 40

Grooming-by-proficiency interactions and simple slopes.

Equation and quantity	b	HC3 SE	t	p	95% HC3 CI	ΔR²	f^2
Pride: grooming x proficiency	.070	.023	3.03	.003	[.024, .115]	.009	.014
Bridging: grooming x proficiency	.035	.023	1.54	.125	[−.010, .080]	.002	.004
Identity: grooming x proficiency	.033	.013	2.50	.013	[.007, .058]	.002	.010
Pride: grooming slope at −1 SD proficiency	.372	.043			[.288, .456]		
Pride: grooming slope at mean proficiency	.460	.031			[.400, .520]		
Pride: grooming slope at +1 SD proficiency	.548	.042			[.466, .630]		
Bridging: grooming slope at −1 SD proficiency	.574	.042			[.491, .656]		
Bridging: grooming slope at mean proficiency	.618	.028			[.564, .673]		
Bridging: grooming slope at +1 SD proficiency	.663	.038			[.588, .738]		
Identity: grooming slope at −1 SD proficiency	.185	.028			[.130, .241]		
Identity: grooming slope at mean proficiency	.227	.022			[.183, .271]		
Identity: grooming slope at +1 SD proficiency	.268	.027			[.215, .321]		

Note. Proficiency and grooming were mean-centred. Simple slopes are the grooming coefficients one standard deviation below the mean, at the mean and one standard deviation above the mean of proficiency, with HC3 standard errors.

10.5 Effect sizes for all interaction terms

Appendix Table 41

Cohen's f^2 for the interaction terms.

Interaction	b	ΔR²	f^2	Benchmark (Cohen, 1988)
Grooming x uniqueness on pride (H4)	.044	.006	.010	below Cohen's small (.02)
Grooming x uniqueness on bridging capital (exploratory)	−.037	.004	.007	below Cohen's small (.02)
Grooming x proficiency on pride	.070	.009	.014	below Cohen's small (.02)
Grooming x proficiency on bridging capital	.035	.002	.004	below Cohen's small (.02)
Grooming x proficiency on identity	.033	.002	.010	below Cohen's small (.02)

Note. ΔR² is the incremental variance explained by the interaction term over the same equation without it; f^2 = ΔR² / (1 − R² of the full equation). Cohen's benchmarks: .02 small, .15 medium, .35 large.
